# Correlative nanoscale chemical imaging reveals delocalized Faradaic processes at isolated MXene flakes

**DOI:** 10.1038/s41467-026-79237-7

**Published:** 2026-11-09

**Authors:** Louis Godeffroy, Lua Henderson, Marc Brunet Cabré, Christian Schröder, Zoé Dessoliers, Namrata Sharma, Kim McKelvey, Paula E. Colavita, Tristan Petit

**Affiliations:** 1 Nanoscale Solid-Liquid Interfaces, Helmholtz-Zentrum Berlin für Materialien und Energie GmbH, Berlin, Germany; 2 School of Chemistry, Trinity College Dublin, College Green, Dublin 2, Ireland; 3 MacDiarmid Institute for Advanced Materials and Nanotechnology and School of Chemical and Physical Sciences, Victoria University of Wellington, Wellington, New Zealand

**Keywords:** Electrochemistry, Two-dimensional materials, Imaging studies

## Abstract

Two-dimensional transition metal carbides and nitrides (MXenes) combine an electrochemically active surface with high conductivity and hydrophilicity, which makes them a unique class of materials for electrochemical applications. While the pseudocapacitive behaviour of MXenes in acidic environments is relatively well understood on macroscale electrodes, intrinsic electrochemical phenomena occurring on single MXene flakes remain unclear. Here, we use scanning X-ray microscopy (SXM) to correlate the electronic structure of Ti_3_C_2_T_*x*_ MXene flakes at the nanoscale with local electrochemical reactions triggered at the sub-flake level by scanning electrochemical cell microscopy (SECCM). For several proton-coupled electron transfer reactions, including electrochemical oxidation and the hydrogen evolution reaction, we demonstrate a delocalization of the reactions well beyond the MXene flake area in contact with the droplet cell. Furthermore, we show evidence of hydrogen storage on monolayer MXene flakes. This work highlights fundamental insights into the role of interfacial water on surface redox reactions.

## Abstract (web summary)

The authors employ a nanoscale chemical imaging technique to image local electrochemical reactions triggered on single MXene flakes. For several electrochemical reactions, a delocalization of the reactions well beyond the MXene flake area in contact with the droplet cell is demonstrated.

## Introduction

Over the last decade, driven by the large-scale deployment of electric vehicles, the energy storage landscape has increasingly shifted toward fast-charging devices. In this context, two-dimensional (2D) materials have attracted considerable attention, as they offer clear advantages over conventional 3D materials by mitigating issues such as limited active surface area and slow ion transport^1^. Among the 2D materials explored to date, transition metal carbides and nitrides, known as MXenes, have shown the greatest promise due to their high conductivity and ultrafast intercalation kinetics. This is particularly exemplified by Ti_3_C_2_T_*x*_ (T_*x*_ representing surface terminations) MXene^2–4^. The intercalation kinetics in MXenes have been shown to strongly depend on surface terminations, with O-terminated Ti_3_C_2_T_*x*_ MXene exhibiting the best performance for H^+^ intercalation by promoting confined water molecules within the interlayers^5,6^. However, this O-terminated Ti_3_C_2_T_*x*_ is also its least stable form, and is prone to spontaneous oxidation to TiO_2_, which results in a complete loss of functional properties^7,8^. It remains unclear whether this oxidation—a major bottleneck for scale-up—can occur or might even be favoured under certain electrochemical conditions.

The oxidation mechanism is not the only unresolved aspect of MXene behaviour. In early studies employing Ti_3_C_2_T_*x*_ MXene as electrode material for pseudocapacitors in acidic media, a striking observation challenged conventional understanding: when comparing planar electrodes to macroporous templated ones, the former—despite their limited accessibility to the electrolyte due to flake restacking—exhibited higher gravimetric capacitances and faster charge-discharge rates^4^. This contradicted the prevailing assumption that increasing the accessible surface area is key to improving capacitance and rate capability. Instead, these results pointed to an alternative mechanism, likely lateral 2D proton transport along the MXene surface^5^. Such surface-confined proton diffusion would be favoured by the dense, well-stacked arrangement of large flakes. While electrochemical impedance spectroscopy (EIS) hinted at such a mechanism, isolating its contribution from bulk electrode effects remains a major challenge. Moreover, in such thick MXene electrodes, it is impossible to fully decouple intercalation from redox pseudocapacitance.

To disentangle the intrinsic electrochemical behaviour of individual flakes, we recently turned to scanning electrochemical cell microscopy (SECCM). By probing small (∼300 nm radius) localized regions of monolayer MXene flakes under conditions comparable to those used in bulk studies, we observed anomalously high capacitance values when normalized to the probe contact area—exceeding those of state-of-the-art devices^9^. In contrast, when normalized to the total flake area, the capacitance closely aligned with them. This strongly indicates the presence of a highly efficient, long-range proton diffusion pathway across the MXene surface, despite the absence of visible wetting beyond the immediate probe contact area in *post-mortem* scanning electron microscopy (SEM) images.

This delocalised charging of MXene flakes is reminiscent of a previously reported observation of delocalised electrochemistry during SECCM measurements, where isolated Pt nanoparticles located microns away from the droplet cell still participated in electrochemical reactions^10,11^. In these studies, *operando* optical microscopy and *post-mortem* SEM imaging revealed the presence of a thin (∼5 nm) electrolyte film extending from the droplet, forming a tenuous ionic bridge. Under reaction conditions, steep concentration gradients developed, drawing water from the droplet towards the nanoparticles. Their subsequent swelling was linked to the ionic current passing through this thin electrolyte layer. These findings underscore the critical role of thin interfacial water layers in mediating electrochemical connectivity over micron-scale distances.

The understanding of such delocalized electrochemistry remains limited. While optical techniques provide high temporal resolution and are suited to *operando* studies, they often lack the spatial or chemical resolution necessary to unambiguously track local oxidation state changes in materials that are not strongly electrochromic^12–17^. Conversely, SEM offers high spatial resolution but also limited chemical sensitivity. To bridge this gap, scanning X-ray microscopy (SXM) appears as a promising alternative. SXM is a synchrotron-based imaging technique that uses a focused X-ray beam to raster across the sample. The energy tunability of the synchrotron source enables chemical contrast by targeting specific electronic transitions. Moreover, surface and bulk chemical signatures can be disentangled using different detection modes—total electron yield (TEY) and transmission, respectively. This correlative approach has already proven highly effective for probing structural and chemical transformations in catalysts and battery materials at the nanoscale^18–24^.

In this study, we achieve nanoscale chemical imaging of individual Ti_3_C_2_T_*x*_ flakes using SXM following localized electrochemical probing by SECCM. Several reactions such as the hydrogen evolution reaction (HER), capacitive charging and electrochemical oxidation were investigated. SECCM allows us to obtain the electrochemical response of flake sub-domains, thereby disentangling extrinsic contributions that may originate from MXene electrode morphology from intrinsic ones coming only from the MXene flake. By mapping the Ti oxidation state with SXM, we show that—regardless of the reaction under investigation—redox activity extends well beyond the immediate contact point. This correlative approach allows the chemical imaging of delocalized electrochemical processes on MXene flakes. Our experiments further suggest that interfacial water molecules play an important role in mediating fast proton transport across the MXene surface. Beyond this observation of delocalized electrochemistry, we provide insights into the mechanisms governing both cathodic and anodic electrochemical processes, including the possibility of storing hydrogen in the MXene. Because this delocalized mechanism dictates the reactivity of Ti_3_C_2_T_*x*_ in both regimes, we believe it may have implications across an even broader range of applications, whenever proton-coupled electron transfer reactions are involved.

## Results

### Correlative SECCM-SXM

Correlating localized electrochemical activity with chemical state changes at the nanoscale requires an imaging platform compatible with both SECCM and SXM. A substrate previously developed in our group for correlative nanoelectrochemistry enables localized electrochemical measurements via SECCM while supporting SXM imaging in total electron yield (TEY) mode on the same nano-entities^25^. It consists of a thin layer of amorphous carbon deposited atop a SiN_x_ membrane. The resulting substrate is electrically conductive, optically and X-ray transparent, chemically homogeneous and inert over millimetre-scale areas, and exhibits an RMS surface roughness below 1 nm. The TEY-SXM mode is particularly advantageous for characterizing surface reactions due to its shallow probing depth of 2-4 nm at the Ti L-edge, mostly probing the first MXene layer (3 Ti layers), compared to the bulk-sensitive transmission mode (STXM)^26^. By combining TEY and transmission measurements, surface chemical changes can be disentangled from thickness variations.

**Fig. 1:**
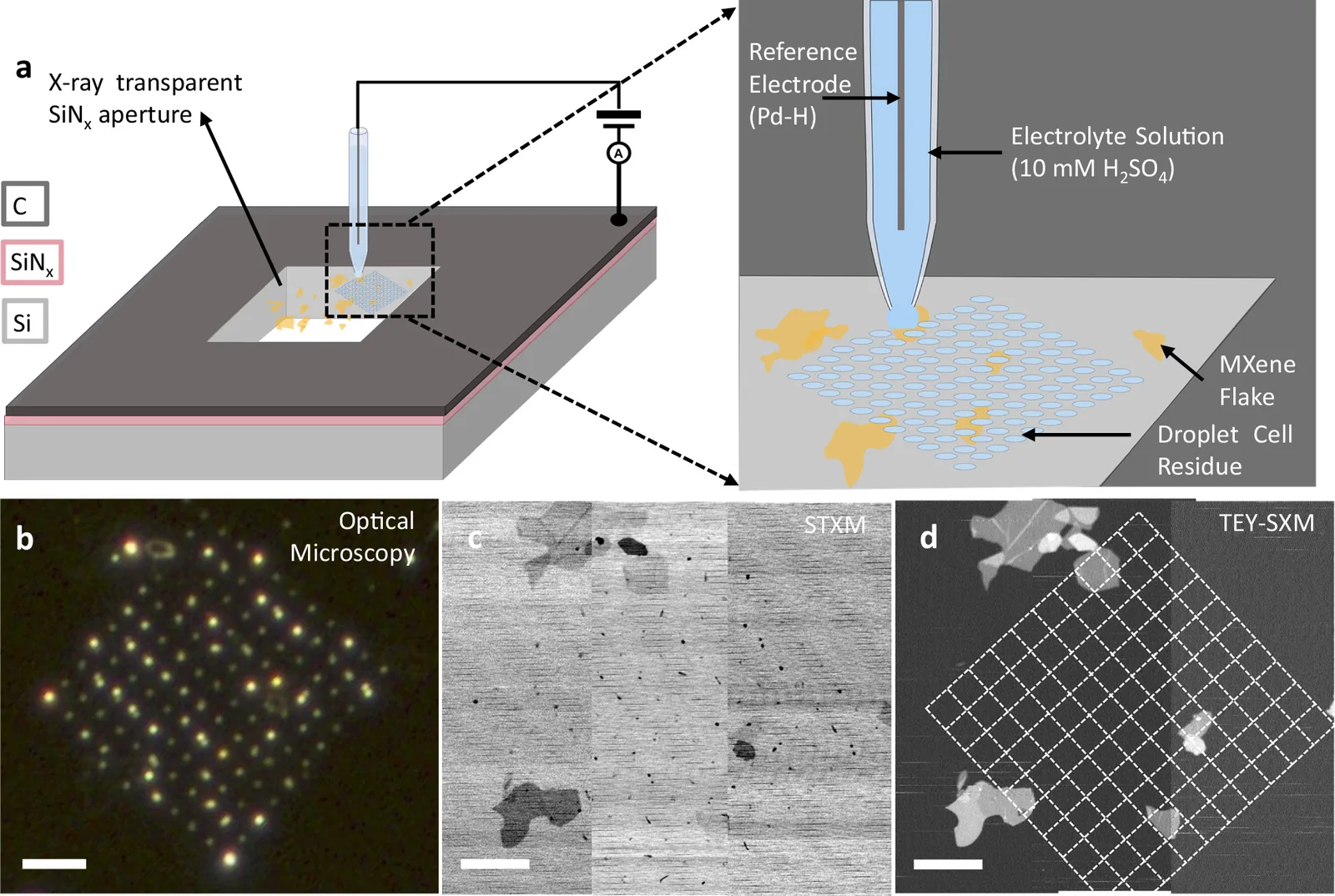
Experimental workflow. **a** Schematic of the SECCM configuration used to measure the electrochemical response of individual MXene flakes on the supporting carbon film (ca. 80 nm thick) deposited on a SiN_x_ window (100 nm thick). **b** Dark-field optical image of the full SECCM grid. The bright spots mainly correspond to the electrolyte residues left behind after a region was probed with the nanopipette (droplet cell residue ∼0.5 µm^2^). **c** Stitched scanning transmission X-ray microscopy (STXM) and **d** stitched total electron yield-scanning X-ray microscopy (TEY-SXM) images of the full SECCM grid acquired at 463 and 465.3 eV, respectively. TEY-SXM image includes added dashed lines to indicate grid point positions in relation to MXene flakes. The stitched images acquired at 530 eV (O K-edge) are provided in Supplementary Fig. 1. Scale bars: 10 µm.

As illustrated in Fig. 1, which provides an overview of the experimental workflow, dilute dispersions of Ti_3_C_2_T_*x*_ MXene flakes were drop-cast onto the substrate (see Methods for details), resulting in individual or grouped flakes dispersed across the surface. SECCM measurements were performed using pipette probes with an aperture radius of *r*_*ap*_ ≈ 0.4 µm and a Pd–H quasi-reference counter electrode in 10 mM H_2_SO_4_ (Fig. 1a). Multiple randomly selected sample regions were probed with grid patterns of 11 × 11 points. At each point, three consecutive cyclic voltammograms were recorded. The positions of the SECCM grids over the sample were subsequently identified using optical microscopy (Fig. 1b), enabling rapid location of the SECCM-probed regions by SXM. Some grids were probed under cathodic conditions (covering the HER and capacitive range), while others—such as the one shown in Fig. 1b–d—were subjected to anodic potentials to induce irreversible oxidation of the MXene flakes^27,28^.

### Anodic cycling

**Fig. 2:**
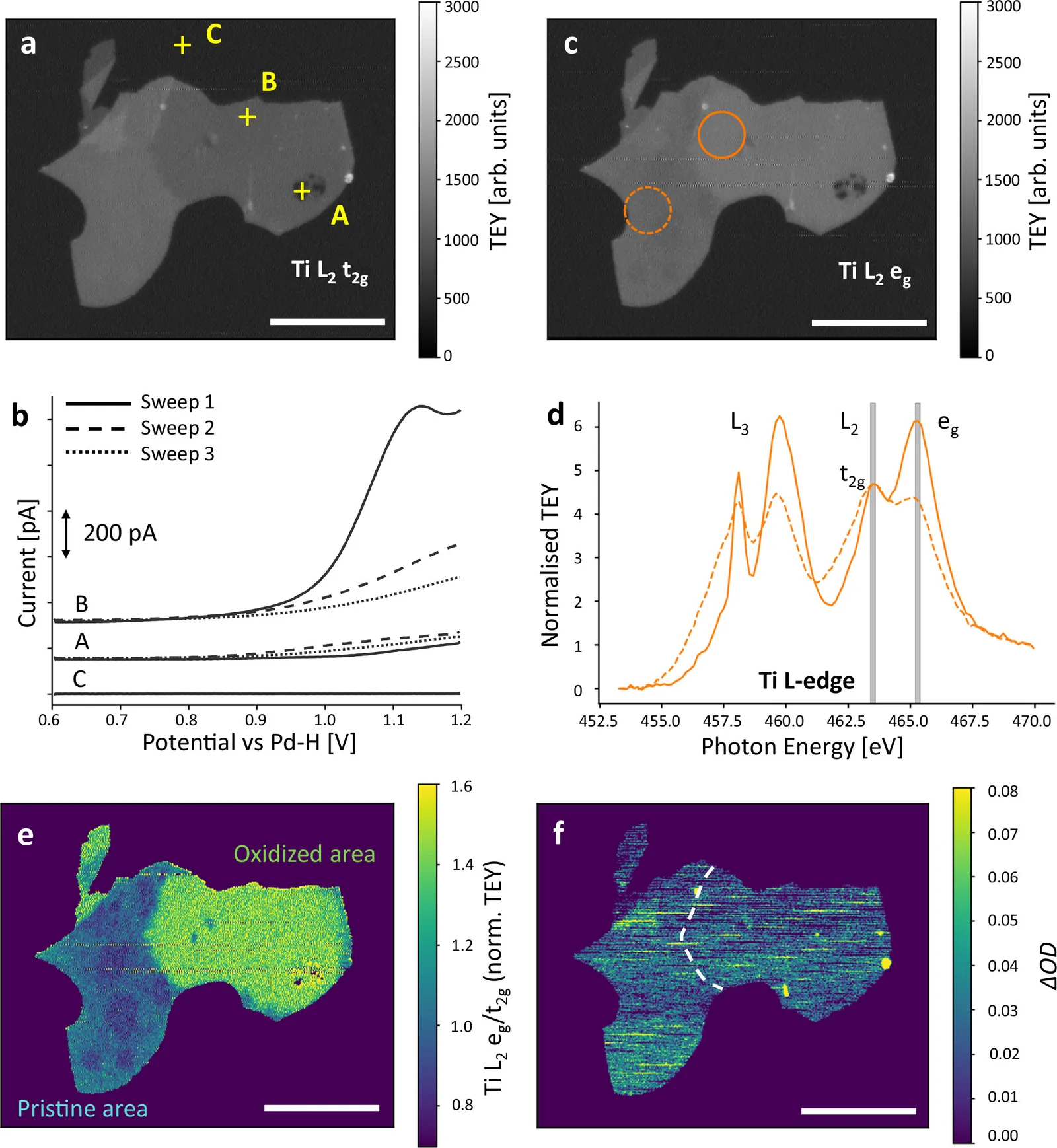
Anodic cycling of a monolayer Ti_3_C_2_T_x_ MXene flake*.* **a** TEY-SXM image acquired at 463.5 eV (Ti L_2_ t_2g_ peak energy). SECCM contact positions are marked with crosses. **b** Anodic sweeps recorded at the SECCM contact points indicated with capital letters in (**a**) at a scan rate of 0.5 V s^−^^1^ in 10 mM H_2_SO_4_. Sweeps at contact points A and B have been offset by 150 and 300 pA, respectively. **c** TEY-SXM image acquired at 465.3 eV (Ti L_2_ e_g_ peak energy). **d** TEY-XAS spectra at the Ti L-edge collected in the regions highlighted in orange in (**c**). The corresponding transmission spectra are provided in Supplementary Fig. 3a. **e** Ti L_2_ e_g_/t_2g_ peak ratio heatmap calculated from the normalized TEY images (details of the image analysis are given in Methods). The corresponding heatmap derived from the transmission images is provided in Supplementary Fig. 3b. **f** STXM image acquired at 463.5 eV (Ti L_2_ t_2g_ peak energy); absorption is shown as optical density (*OD*) normalised to the pre-edge. We show in Supplementary Note 1 that, for thicknesses below ∼40 layers, *OD* scales linearly with thickness. Scale bars: 5 µm.

The effect of anodic cycling (0–1.2 V vs Pd-H) on the surface chemistry of a monolayer Ti_3_C_2_T_*x*_ MXene flake contacted by the SECCM probe is shown in Fig. 2. The exact SECCM contact positions (denoted by capital letters in Fig. 2a) were identified based on stitched STXM images of the entire grid (Fig. 1c, d), which display electrolyte residues left upon droplet retraction. The three anodic sweeps collected at each of the three SECCM contact points are presented in Fig. 2b, while the complete set of cyclic voltammograms is provided in Supplementary Fig. 2. X-ray absorption spectra (XAS) both near and far from the contact points (regions highlighted in Fig. 2c) were collected at the Ti L-edge (Fig. 2d). From these spectra, the Ti oxidation state can be inferred using either the L_3_/L_2_ intensity ratio or the e_g_/t_2g_ ratio at the L_3_ or L_2_ edge^29^. Applying this analysis pixel by pixel, we generated a spatial map of the L_2_ e_g_/t_2g_ ratio (Fig. 2e), which reveals two distinct regions across the flake. The area surrounding the SECCM contact points (approximately 41 µm^2^, compared with the total flake area of 71 µm^2^) exhibits a markedly higher oxidation state (∼4.4 based on the calibration curve in Supplementary Fig. 3 and according to ref. ^29^), while regions farther away remain less oxidized (∼3.0). Importantly, the oxidized region extends well beyond the immediate vicinity of the contact points, demonstrating the delocalized nature of the anodic redox process. Notably, the pristine region exhibits a slightly higher oxidation state than freshly synthesized MXene^26^, most likely due to spontaneous oxidation upon exposure to humid air^30^.

Crucially, this oxidation front cannot be attributed to variations in flake thickness. Transmission-mode STXM confirms that the flake exhibits a uniform thickness across its entire area and based on its average optical density (0.024), we estimate it to be a monolayer (Fig. 2f and Supplementary Fig. 4). This rules out thickness as a primary source of the observed heterogeneity, thus reinforcing that the extended redox changes are electrochemically driven.

The anodic sweeps obtained following contact at the MXene sub-domains (A and B, Fig. 2b) confirmed that an irreversible oxidation process takes place over the 0.8–1.2 V potential window. Interestingly, most of the anodic charge was transferred during the second contact (B) of the flake. As shown in Supplementary Fig. 5, this priming effect appears to be a general behaviour across most studied flakes. In contrast, anodic sweeps at points contacting the carbon support (e.g., point C in Fig. 2b) were highly reproducible and involved much lower anodic currents, consistent with double-layer capacitive charging alone (Supplementary Figs. 6 and 7). The faradaic charges passed after contacting the MXene sub-domains were 178 pC (point A) and 769 pC (point B, Supplementary Fig. 8), values much larger than expected for complete oxidation of a Ti_3_C_2_T_*x*_ monolayer over the ∼0.5 µm^2^ area defined by the probe aperture (∼20 pC, calculation details in Supplementary Note 2). This behaviour was consistently observed across all SECCM grid points contacting MXene flakes, as shown in the heat maps of faradaic anodic charge in Supplementary Fig. 9. These results indicate that the oxidation process must extend over an area of the MXene flake much larger than that defined by the nanopipette aperture, as suggested by the chemical imaging of the Ti oxidation state in Fig. 2e.

On the other hand, the total oxidation charge passed at the MXene flake, 947 pC, closely matches the total charge expected for an 11-electron process over an area of 43 µm^2^, i.e., in excellent agreement with the 41 µm^2^ region of high oxidation state shown in Fig. 2e. The total number of electrons involved in Ti_3_C_2_T_*x*_ oxidation depends on the products formed and the fraction *x* of surface terminations. For oxidation yielding TiO_2_ and elemental carbon^28,31–33^, the following equation can be proposed:

1 $$T{i}_{3}{C}_{2}{T}_{x}+6{H}_{2}O\longrightarrow 3{Ti}{O}_{2}+2C+12{H}^{+}+\left(12 - x\right)e+x{T}^{ - }$$

This corresponds to a 10–11-electron process for *x* = 1–2, in agreement with previously reported Ti_3_C_2_T_*x*_ stoichiometries.

This result demonstrates that the combination of SECCM with SXM mapping enables to assess how far the reaction front propagates upon application of a localized electrochemical stimulus. Interestingly, a large fraction of the flake is oxidised in our experiments (∼58%), compared with estimates of %Ti oxidised using the nano-impact method by Compton and co-workers (<3%)^34^. This suggests that the progression of the oxidation front occurs over relatively long timescales, which may be facilitated by the stable contact provided by the SECCM configuration, in contrast to the short timescales inherent to nano-impact measurements in solution.

Notably, this electrochemical oxidation process appears different from spontaneous oxidation: even over longer timescales (hours), the spontaneous oxidation of single Ti_3_C_2_T_*x*_ flakes was found to be reversible upon switching between water and H_2_SO_4_, based on our recent in situ STXM measurements^29,30^. This observation further supports the irreversible formation of TiO_2_ under electrochemical conditions, as proposed above. Electrochemical oxidation therefore seems to lead to a similar material as oxidation in air at high temperature^35^. Complementary TEY-XAS measurements at the C K-edge on thick MXene electrodes also confirm the loss of the MXene structure following electrochemical oxidation (see Supplementary Note 3 and Supplementary Fig. 10).

**Fig. 3:**
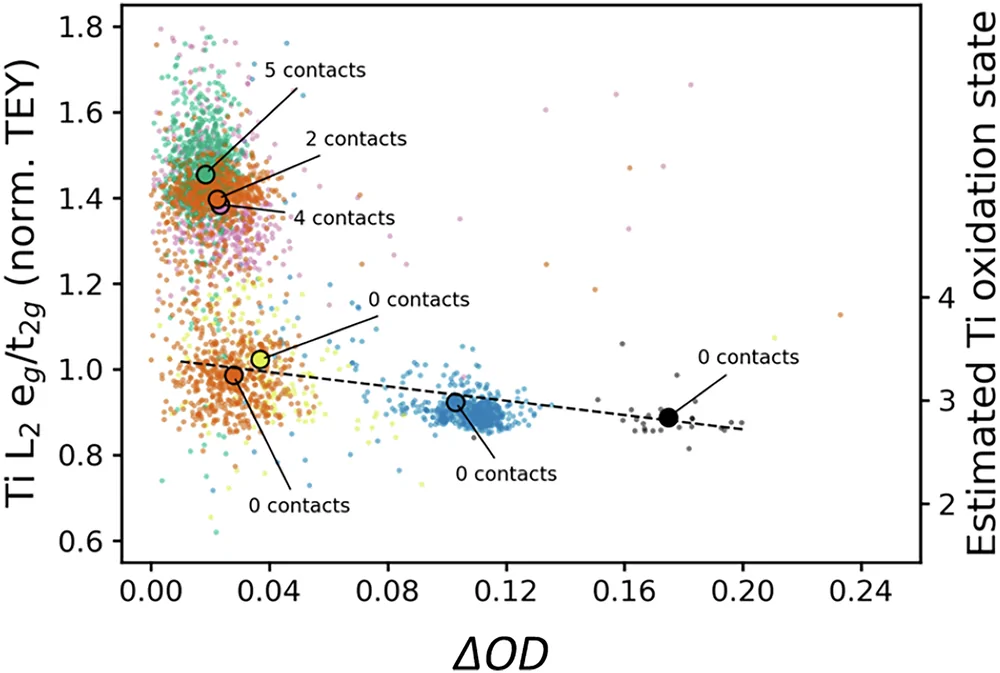
Statistical analysis across multiple Ti_3_C_2_T_x_ MXene flakes*.* Pixel-by-pixel scatter plot of estimated Ti oxidation state (Ti L_2_ e_g_/t_2g_ ratio) vs *ΔOD* for flakes cycled under anodic conditions. The Ti oxidation state was estimated based on the calibration curve presented in Supplementary Fig. 3 and according to ref. ^29^. Corresponding flake images, from different grids, are provided in Supplementary Fig. 11, and details of the image analysis are given in Methods. Only the pixels corresponding to MXene flakes were considered here. The larger dots correspond to each distribution’s centre of mass.

To gain statistical insight into this anodic process, a similar analysis of the STXM images at the Ti L-edge was performed on five additional Ti_3_C_2_T_*x*_ MXene flakes with different sizes, thicknesses, and numbers of SECCM contact points. In Fig. 3, the estimated Ti oxidation state (Ti L_2_ e_g_/t_2g_ ratio) is plotted against flake thickness (optical density) on a pixel-by-pixel basis.

A significant increase in Ti oxidation state is observed only for contacted flakes, confirming the electrochemical origin of the oxidation. The bimodal distribution shown in orange corresponds to the flake discussed in Fig. 2, which exhibits two distinct regions with different oxidation states at the same thickness. Comparing these results to flakes of similar thickness cycled in the cathodic range (Supplementary Fig. 12), which also show oxidation but to a lesser extent (∼3.9 vs 4.4), allows spontaneous oxidation to be ruled out even more convincingly. In both cases, among non-contacted flakes, thinner ones exhibit a greater tendency to oxidize spontaneously—presumably due to their higher proportion of surface Ti atoms exposed to humid air. While this behaviour may seem intuitive, that the top layer protects the ones below from oxidizing, this represents a direct experimental demonstration that thinner MXene flakes are more susceptible to spontaneous oxidation than thicker counterparts. Interestingly, Supplementary Fig. 12 also shows that this spontaneous oxidation process becomes exponentially more pronounced for thicknesses below 2–3 layers, consistent with a recent imaging ellipsometry study of the resistivity of individual Ti_3_C_2_T_*x*_ flakes^36^. This insight could help guide the rational design of more stable MXenes for future applications.

### Cathodic cycling

**Fig. 4:**
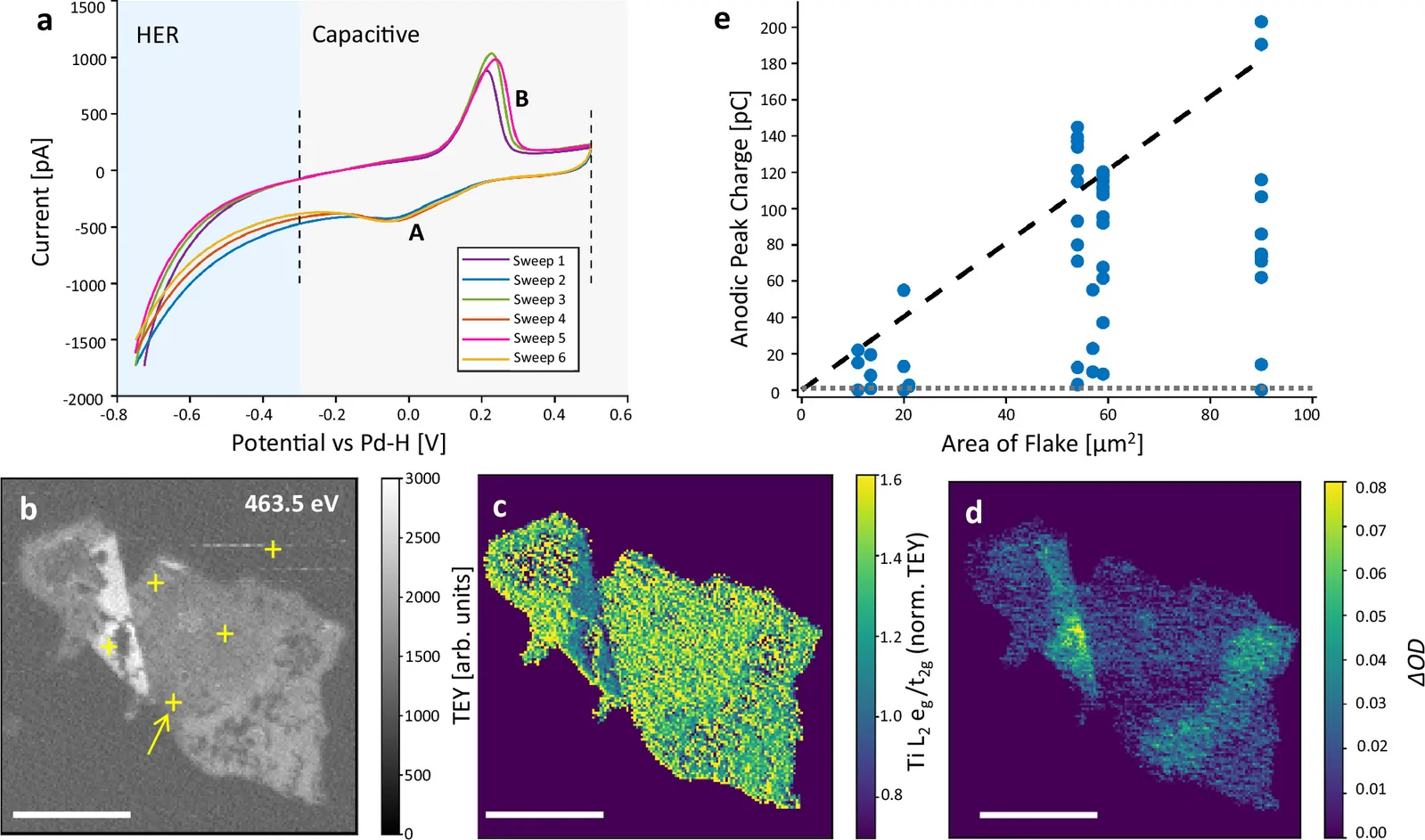
Cathodic cycling of a monolayer Ti_3_C_2_T_x_ MXene flake across the HER and capacitive potential windows*.* **a** Cyclic voltammograms measured at a scan rate of 0.5 V s^−^^1^ in 10 mM H_2_SO_4_ obtained at the location indicated by an arrow in (**b**); the cathodic and anodic peaks discussed in the text are labelled A and B, respectively. **b** TEY-SXM image acquired at 463.5 eV (Ti L_2_ t_2g_ peak energy). SECCM contact positions are marked with crosses. **c** Ti L_2_ e_g_/t_2g_ peak ratio heatmap calculated from the normalized TEY images (details of the image analysis are given in Methods). The corresponding heatmap derived from the transmission images is provided in Supplementary Fig. 18. **d** STXM image acquired at 463.5 eV (Ti L_2_ t_2g_ peak energy); absorption is shown as optical density (*OD*) normalised to the pre-edge. **e** Anodic peak charges (peak B) extracted from all SECCM grids. Dashed line (black) indicates expected peak charge for a 1-electron process per unit cell. Dotted line (grey) indicates expected peak charge values for a 1-electron process if the process was confined to the contact area probed by the nanopipette, ∼0.5 µm^2^ (Supplementary Fig. 15). Scale bars: 5 µm.

In addition to probing the anodic response, we also investigated the cathodic behaviour by measuring SECCM grids between −0.75 V and 0.5 V, thus covering both the HER (−0.75 V to −0.3 V) and the capacitive potential window (−0.3 V to 0.5 V, Fig. 4a). As before, three consecutive cycles were collected at each grid point. The cyclic voltammograms exhibit reversible responses at both MXene- or carbon-contacting positions (Fig. 4a and Supplementary Fig. 13), although the HER currents at the cathodic limit of the potential window are significantly larger at MXene-contacting points (over 300 times larger, Supplementary Fig. 14). The overall cathodic activity is also markedly higher than in previous reports, most likely due to differences in surface chemistry^37^: the MXenes used here were prepared by HF/HCl etching, in contrast to the MILD-etched (with in-situ generated HF) samples used in ref. ^9^. An example of a flake probed under these conditions (total area: 93.5 µm^2^) is shown in Fig. 4b. The corresponding spatial map of the L_2_ e_g_/t_2g_ ratio (Fig. 4c) reveals a uniform Ti oxidation state across the entire surface. While the average oxidation state is relatively high, it remains lower than that of electrochemically oxidized flakes. STXM imaging further shows that the MXene flake is monolayered (average *ΔOD* of 0.017, Fig. 4d), which likely accounts for the relatively high oxidation state observed in the L_2_ e_g_/t_2g_ ratio heat map, as thinner flakes are generally more susceptible to spontaneous oxidation (Supplementary Fig. 12).

In the capacitive electrochemical potential window, the three cycles in Fig. 4a illustrate the main features observed across all MXene sub-domains probed in SECCM grid experiments (Supplementary Fig. 15). A broad cathodic peak (A, ca. −0.05 V) and a well-defined anodic peak (B, ca. 0.20 V) are observed over a large capacitive background current. The average capacitance of the three cycles in Fig. 4a (excluding contributions from peak B, Supplementary Fig. 16) is 343 pF. Considering the area of the probed monolayer flake and a specific surface area of 272 m^2^ g^−1^^9^ this corresponds to a specific capacitance of 998 F g^−1^ (367 µF cm^−2^), or 1.8 electrons per unit cell per volt (see Supplementary Note 2). Similar capacitances are observed at nearly all MXene-contacting points in our experiments (Supplementary Fig. 17). Such values are too large to arise solely from double-layer charging and protonation/deprotonation processes, which typically yield 150–250 F g^−1^ (up to 0.4 electrons per unit cell per volt)^9,38,39^. However, they are consistent with the MXene operating in the hydrogen storage regime as a result of cathodic polarization extending into the HER region^38,40–44^. Indeed, maximum capacitances of up to 1000-2000 F g^−1^ have been reported when surface terminations are tailored to maximise hydrogen sorption capacity, provided that ion and hydrogen transport limitations remain negligible^4,45,46^. SECCM probing of isolated monolayers appears to achieve such conditions, resulting in gravimetric pseudocapacitances close to the upper limit attainable for a given surface termination.

Supposing that the MXene flake indeed operates in the hydrogen storage regime, peaks A and B in Fig. 4a could be attributed to hydrogen sorption and desorption processes, respectively. The cathodic sorption peak A is centred around potentials comparable to those observed at bulk Ti_3_C_2_T_*x*_ electrodes in sulfuric acid (see also Supplementary Fig. 19)^4,41^. In contrast, peak B appears at potentials 0.2−0.4 V more anodic than the main desorption peak at bulk electrodes, and its peak potential is narrowly distributed around 0.2 V (Supplementary Fig. 20). Our interpretation is that hydrogen desorption at isolated monolayers arises from specific surface sites with a narrow distribution of binding energies. The peak potential of this feature also suggests that these sites exhibit relatively high binding strengths, being 20-40 kJ mol^−1^ more stable (ΔΔG = FΔV) than those associated with the main desorption peak at bulk electrodes.

The integrated charge of peak B varies in magnitude but shows a positive correlation with the size of the contacted MXene flake (Fig. 4e). The electrochemically probed point indicated by the arrow in Fig. 4b has an average desorption charge of 170 pC. Considering the flake area (93.5 µm^2^), this is equivalent to 0.9 electrons per unit cell. The dashed line in Fig. 4e represents the charge expected for a 1-electron process per unit cell. Most desorption charge values fall below this boundary, indicating that the active sites responsible for the anodic peak have a maximum occupancy of 1 (one hydrogen atom per unit cell). This maximum occupancy corresponds to a hydrogen sorption capacity of approximately 0.5 wt%, comparable to the 0.44 wt% achieved with Ti_3_C_2_T_*x*_ in pressure-driven hydrogen storage systems^47^, further supporting our interpretation. The scatter observed in Fig. 4e, especially for larger flakes, likely arises from the fact that only a portion of the flake is addressed during SECCM probing. Although no Ti oxidation state fronts could be detected in the STXM images (the expected changes being smaller than those caused by spontaneous oxidation), the desorption peak charges clearly demonstrate that redox activity extends well beyond the SECCM probe contact area. Indeed, almost all charge values exceed that expected for a 1-electron desorption process confined to the ∼0.5 µm^2^ contact area (ca. 1 pC, horizontal dotted line in Fig. 4e, see also Supplementary Fig. 21). This phenomenon closely mirrors the delocalized electrochemical behaviour observed under anodic cycling. Possible origins of this effect are discussed below.

Recent work by Koudahi and Frackowiak highlighted the influence of different types of adsorption sites on hydrogen desorption at Ti_3_C_2_T_*x*_ electrodes in acid^41^. They proposed that desorption peaks appearing at potentials ≤ 0 V vs SHE originate from weakly bound, physisorbed hydrogen species, whereas those near 0.17 V vs SHE correspond to more strongly bound, chemisorbed species. In the case of isolated flakes, desorption of physisorbed hydrogen would likely be rapid and occur unhindered over a wide range of low potentials, facilitated by high surface mobility and efficient transport to the electrolyte/gas interface. This could explain the absence of a distinct desorption peak in the −0.2 V to 0 V range in our single-entity SECCM experiments. However, desorption from specific high-binding-energy sites would still incur a measurable thermodynamic penalty, giving rise to a well-defined redox peak—consistent with our observations. These results suggest that hydrogen desorption studies at individual MXene flakes are particularly well suited to probe high-energy sites whose contributions are normally masked by physisorption processes at bulk electrodes. Moreover, the agreement between our electrochemical desorption data and pressure-driven hydrogen storage capacities further indicates that both approaches likely probe the same class of hydrogen binding sites.

**Fig. 5:**
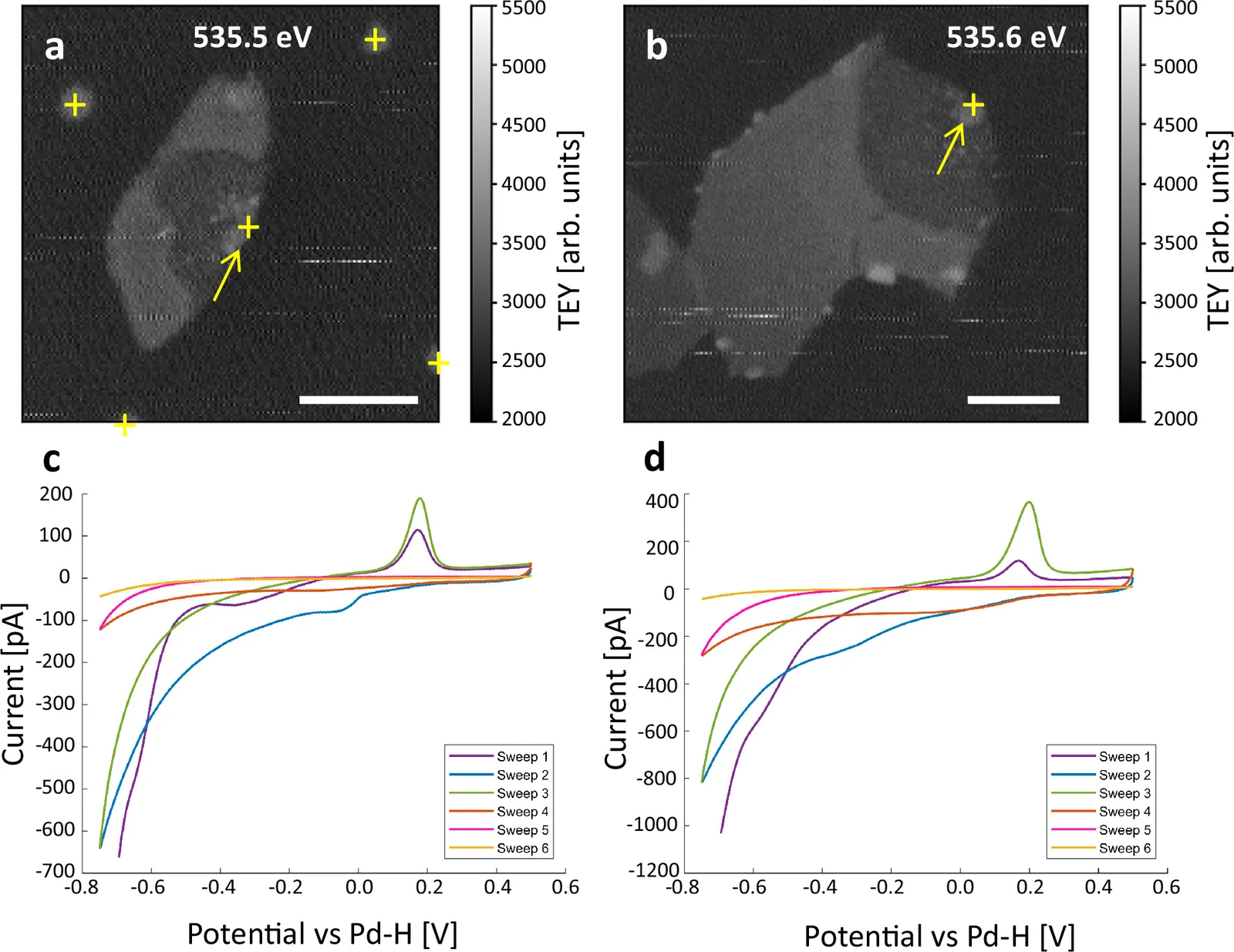
Water depletion at individual Ti_3_C_2_T_x_ MXene flakes after cathodic cycling*.* **a**, **b** TEY-SXM images acquired at 535.5 and 535.6 eV, respectively (interlayer water feature at the O K-edge); positions of electrolyte residues remaining after SECCM probing are visible and indicated by crosses. **c**, **d** Cyclic voltammograms measured at a scan rate of 0.5 V s^−1^ in 10 mM H_2_SO_4_ at the locations marked with arrows in (**a**) and (**b**), respectively. Scale bars: 2 µm.

The experimentally observed reversibility of the HER-pseudocapacitive response is probably underpinned by rapid replenishment of surface hydrogen following H_2_ evolution and H desorption during the cathodic sweep of each cycle. The nanopipette probe can serve as a water and proton reservoir; however, depending on the scan rate, ambient humidity, and surface chemistry, the rate of water or proton reduction may exceed that of their transport across the flake surface. Evidence supporting this scenario is provided by TEY-SXM measurements at both the Ti L-edge and O K-edge on selected flakes (Fig. 5 and Supplementary Fig. 22). In these two examples, the Ti oxidation state is relatively homogeneous across the MXene flake (Supplementary Fig. 22). Although transmission-mode data are not available, the uniformly elevated oxidation state suggests they are very thin—likely fewer than 2 layers (Supplementary Fig. 12). At the O K-edge (Fig. 5a, b), however, a distinct depletion in signal is observed near the SECCM contact points, specifically at 535.5 eV. According to reference spectra of MXene electrodes in Supplementary Fig. 10, this feature does not correspond to changes in the MXene’s surface chemistry. Instead, it has previously been attributed to the pre-edge of the electronic signature of water molecules^30,48^. The localized signal loss at this energy therefore indicates a depletion of intercalated water following cathodic cycling in H_2_SO_4_. This interpretation is consistent with previous infrared spectroscopy studies from our group, which showed that water is partially expelled from the MXene structure both upon simple exposure to H_2_SO_4_ and during capacitive cycling^49,50^. However, the strong HER currents measured here suggest that, in the present case, water may have been consumed irreversibly during hydrogen desorption.

The cyclic voltammograms obtained for these two flakes indeed show a decrease in capacitive current accompanied by a loss in HER activity in the third and final cycle. They are two of only three SECCM points exhibiting this behaviour across all measurements (*N* = 18). As shown in Fig. 5c, d, high HER currents and pseudocapacitances (approximately 40 and 100 pF) are observed during the first two cycles, but both decrease markedly in the third. In fact, the pseudocapacitive background collapses to roughly 5% of its initial value in the final cycle. This result can be interpreted either by the electrochemical response that becomes increasingly localized due to water depletion—no longer extending over the entire area of the flake—or by irreversible changes in the MXene surface chemistry occurring during cycling, leading to a loss of hydrogen storage capacity. Both interpretations are consistent with the depletion of intercalated water inferred from the O K-edge TEY-SXM images. However, these three flakes share the common feature of being relatively small compared to the others. As a result, the remaining flake area might no longer act as an effective water reservoir, leading to strong local depletion in the vicinity of the SECCM contact points.

## Discussion

We show that electrochemical oxidation, capacitive charging, and probably hydrogen desorption all yield delocalized electrochemical responses—that is, responses extending, based on charge measurements and SXM observations, beyond the area directly contacted by the SECCM droplet. Notably, SECCM contact points located on the bare carbon-coated membrane in Fig. 5a do not show any signs of strong wetting, indicating that these delocalized responses are a characteristic of contact with MXenes.

All the investigated reactions share a common feature: they are proton-coupled electron transfer (PCET) processes (see Equations S2-4 in Supplementary Note 3). The fact that each exhibits delocalized electrochemical behaviour thus provides indirect evidence that proton transport across the MXene surface, or within its interlayer spaces, governs the overall response. Due to its mostly oxygenated surface terminations, Ti_3_C_2_T_*x*_ is known to strongly interact with water and protons, creating a favourable environment for proton transport^5,51^. How hydrogen adsorption sites become filled across large portions of the flakes remains unclear at this stage, although most studies of hydrogen storage in MXenes propose formation and storage of molecular hydrogen^41,42,47,52^. The hypothetical storage capacity estimated from our SECCM results appears consistent with prior computational and experimental estimates of H_2_ storage in MXenes.

For both the HER and capacitive charging, the sharp decrease in electrochemical activity that accompanies the irreversible consumption of water during hydrogen desorption in Fig. 5 suggests that proton transport might be mediated by the presence of water within the interlayers or at the flake surface. This observation is reminiscent of a previous report of delocalized electrochemical activity, where a nanometric electrolyte channel was identified as an ionic bridge sustaining the oxygen reduction reaction at Pt nanoparticles located outside the apparent boundary of the electrochemical cell^10,11^.

In conclusion, we have disentangled the intrinsic electrochemical behaviour of individual Ti_3_C_2_T_*x*_ MXene flakes in anodic and cathodic regimes using a correlative nanoscale chemical imaging approach, combining SECCM and SXM. Single-entity approaches are key to decorrelate surface redox from intercalation phenomena in MXenes and in 2D materials in general. The combination of SECCM and SXM enables here the mapping of the reaction front of faradaic processes across the MXene surface with nanoscale resolution. In the anodic regime, we demonstrated that electrochemical Ti_3_C_2_T_*x*_ oxidation leads to the irreversible formation of TiO_2_, while preserving the 2D flake structure. Our results further identify flake thickness as a key factor governing susceptibility to spontaneous oxidation. In the cathodic regime, we identified well-defined redox peaks, which are interpreted as strong hydrogen adsorption sites. These would typically be masked by the broad redox peaks associated with weak physisorption sites in bulk electrodes. Beyond providing a better understanding of the mechanisms at play in the different regimes, we reveal that most of these reactions proceed in a delocalized manner, extending beyond the contact area defined by the SECCM probe aperture. Because all these reactions involve proton-coupled electron transfer, we hypothesize that surface proton transport across the MXene surface is responsible for this delocalized behaviour, underscoring the crucial role of interfacial water in defining the reactivity of 2D materials such as MXenes.

## Methods

### Chemicals

Hydrofluoric acid (HF, 48.5–51%, Sigma-Aldrich), hydrochloric acid (HCl, 36.5–38%, Fisher Chemical), lithium chloride (LiCl, 99%, Acros Organics), and sulfuric acid (H_2_SO_4_, 99.999%, Sigma-Aldrich) were used as received. All solutions were prepared with ultrapure water (>18.2 MΩ, Millipore).

### MXene synthesis

Ti_3_C_2_T_*x*_ MXene was synthesized by selectively etching the Al layer from Ti_3_AlC_2_ MAX phase (provided by Jesus Gonzalez-Julian, LCTS, Bordeaux, France) using a mixture of HF, HCl, and ultrapure water in a volume ratio of 1:6:3, following the procedure reported by Shuck et al.^53^ Specifically, 1 g of MAX phase powder was stirred in 25 mL of the acid mixture for 24 h at 35 °C. The resulting multilayer Ti_3_C_2_T_*x*_ were repeatedly washed with ultrapure water until the pH of the supernatant reached between 5 and 7.

To delaminate the multilayer Ti_3_C_2_T_*x*_, the sediment was dispersed in 50 mL of 0.5 M LiCl aqueous solution and stirred for 18 h at room temperature. The intercalated multilayer Ti_3_C_2_T_*x*_ was then washed multiple times with ultrapure water (150 mL per wash). The first two washes were performed by centrifugation at 3500 rpm (2250 × *g*) for 10 min, while the third wash was extended to 60 min to ensure complete removal of residual Li^+^ ions. From the fourth wash onward, the supernatant became visibly thicker and was collected as a suspension of delaminated Ti_3_C_2_T_*x*_ flakes.

To improve the stability and shelf life of the dispersion, the supernatant was concentrated by centrifugation at 5000 rpm (4580 × *g*) in 60 mL tubes, and the sedimented Ti_3_C_2_T_*x*_ flakes were collected and transferred to a storage vial. The vial was flushed with Ar and stored at 5 °C until further use to minimize oxidation. An X-ray diffraction pattern and scanning electron micrograph confirming the successful synthesis are shown in Supplementary Fig. 23.

### Preparation of the carbon-coated SiN_x_ membranes

Carbon deposition was performed on Si/SiN_x_ substrates with SiN_x_ windows (Norcada) using sputtering deposition methods as previously reported^54^. Sputtering was carried out in a DC magnetron sputtering chamber (Torr International, Inc.) using a graphite target (99.999%, Kurt J. Lesker) at a base pressure of <2 × 10^−6^ mbar for 40 min under an inert atmosphere (Ar 50 sccm, 1–2 × 10^−2 ^mbar). Films were then annealed at 900 °C in a tube furnace (Carbolite Gero) under N_2_ flow (200 sccm, 60 min), yielding carbon electrodes of thickness 73 ± 3 nm as previously reported^55^.

The concentrated Ti_3_C_2_T_*x*_ flakes were diluted by a factor of 500 and subsequently drop-cast (2 µL) on the prepared carbon electrodes within 24 h of obtaining the stock solution. Samples were left to dry overnight in air, obtaining regions within the drop-cast area with single MXene flakes on the carbon substrate. All electrochemical measurements were carried out within 1 day of drop casting. The samples were then sealed in a nitrogen atmosphere for transport to BESSY II.

### SECCM

Single-barrelled nanopipettes were used as probes for SECCM. These were fabricated from borosilicate capillaries (1.5 mm O.D. and 0.86 mm I.D., BF150-86-7.5, Sutter Instrument, USA) using a P-2000 laser puller (Sutter Instrument, USA). A pipette filler (MicroFil MF34G-5, World Precision Instruments, USA) was used to introduce the electrolyte (10 mM H_2_SO_4_). The quasi-reference counter electrode (QRCE) used for these experiments was a Pd-H electrode. The Pd-H was fabricated from a Pd wire (0.25 mm diameter, PD005130, Goodfellow, UK) by biasing it at −3 V vs a Pt counter electrode for 15 min in acid solution (20 mM H_2_SO_4_) and used for experimentation immediately after^9,56^.

A custom-built electrochemical scanning probe microscope was used to perform SECCM as previously reported^57^. A transimpedance amplifier (DDPCA-300, FEMTO, Germany) was used for all experiments. Hopping mode was employed, allowing the mapping of electrochemical response across predefined grid points (11 ×11 spaced 4 µm apart). Grids were positioned at random locations across the 1 mm^2^ aperture of the SiN_x_ window (see Fig. 1) to allow for subsequent SXM imaging. At each SECCM sample point, three cyclic voltammograms were measured at 500 mV s^−1^ (+1.2 to 0 V for anodic cycling and −0.75 to +0.5 V for cathodic cycling). Hopping mode involved vertically lowering the probe towards the sample at a speed of 0.5 µm s^−1^ until contact was established between the nanopipette droplet and the surface of the sample, as detected by an increase in current. The current threshold was set to 10 pA, so contact was established once this was exceeded. Once the CVs were recorded, the pipette was retracted and moved to the next pre-defined grid point. Samples were then imaged with an optical microscope in bright- and dark-field modes (Olympus DSX1000 digital microscope) to record the approximate location of SECCM grids on the SiN_x_ window. Voltammetry datasets were analysed using custom MATLAB routines.

### SXM

Scanning X-ray microscopy was performed at the MAXYMUS endstation of the UE46-PGM2 undulator beamline at the BESSY II synchrotron (Berlin, Germany). Measurements were conducted under vacuum in both conventional transmission mode (STXM) and total electron yield (TEY) mode. In TEY mode, the sample current was amplified using a commercial current amplifier (FEMTO Messtechnik GmbH, Berlin, Germany) and subsequently converted into a frequency signal. To prevent electrons emitted by the Fresnel zone plate (lens) from reaching the sample, the order-sorting aperture (OSA)—a pinhole that blocks the unfocused zero-order light—was biased at approximately +30 V relative to the sample.

All images were processed using custom Python routines. The TEY images—showing higher contrast for the thin flakes studied—were first aligned using a cross-correlation algorithm. To generate the Ti L_2_ e_g_/t_2g_ peak ratio heat maps (as in Fig. 2e), the two corresponding images were divided pixel by pixel after subtracting the pre-edge image from both, ensuring that thickness effects were excluded from the determination of the Ti oxidation state.

For the ΔOD heat maps (as in Fig. 2f), the TEY-derived x-y shifts were first applied to the transmission images, which were then converted to optical density and corrected by subtracting the pre-edge image (also converted to optical density) from each. This step ensures that any contributions from electrolyte residues potentially left by the pipette are removed. The image acquired at the t_2g_ peak energy was used as a proxy for thickness, as this energy provides maximal contrast while remaining relatively insensitive to the Ti oxidation state.

To produce the plots in Fig. 3, the Ti L_2_ e_g_/t_2g_ peak ratio and ΔOD heat maps were first binned (5×5) to reduce noise and then compared on a pixel-by-pixel basis. Two example scripts for the flake shown in Fig. 2 are provided in a Zenodo repository (https://doi.org/10.5281/zenodo.22283933) along with all raw data.

## Supplementary information

Supplementary Information

Transparent Peer Review file

## Source data

Source Data 1

Source Data 2

## Data Availability

Source data for the graphs presented in the figures are provided in the Source Data file. For the images, the raw data and a set of example scripts to analyse them are available in a Zenodo repository (https://doi.org/10.5281/zenodo.22283933). Source data are provided with this paper.
