# Supplementary material for: Correlative nanoscale chemical imaging reveals delocalized Faradaic processes at isolated MXene flakes
Source: Nat Commun. 2026 Nov 9;17:11330. doi: 10.1038/s41467-026-79237-7 (PMC13748615; doi:10.1038/s41467-026-79237-7)
Supplement: Supplementary file 1 [file 41467_2026_79237_MOESM1_ESM.pdf]

Supplementary Information

Correlative Nanoscale Chemical Imaging Reveals Delocalized Faradaic Processes at Isolated MXene Flakes

Louis Godeffroy,[1,†] Lua Henderson,[2,†] Marc Brunet Cabré,[2] Christian Schröder,[2] Zoé Dessoliers,[1] Namrata Sharma,[1] Kim McKelvey,[3,*] Paula E. Colavita,[2,*] Tristan Petit[1,*]

[1]Nanoscale Solid-Liquid Interfaces, Helmholtz-Zentrum Berlin für Materialien und Energie GmbH, 12489 Berlin, Germany
[2]School of Chemistry, Trinity College Dublin, Dublin 2, Ireland
[3]MacDiarmid Institute for Advanced Materials and Nanotechnology, School of Chemical and Physical Sciences, Victoria University of Wellington, Wellington 6012, New Zealand
[†]Contributed equally
*Corresponding authors: kim.mckelvey@vuw.ac.nz; colavitp@tcd.ie; tristan.petit@helmholtz-berlin.de

Table of Contents

Supplementary Fig. 1. Composite scanning X-ray microscopy (SXM) images obtained at the O K-edge. 3

Supplementary Fig. 2. Complete set of SECCM voltammograms in the anodic region. 4

Supplementary Fig. 3. Additional data relative to Fig. 2. 5

Supplementary Note 1. Optical density of a monolayer of $Ti_3C_2T_x$ 6

Supplementary Fig. 4. Optical density of a monolayer of $Ti_3C_2T_x$. 6

Supplementary Fig. 5. Priming effect. 7

Supplementary Fig. 6. Expanded plot of anodic and cathodic potential sweeps at carbon. 8

Supplementary Fig. 7. Heat map of the integrated charge of the first anodic sweep. 9

Supplementary Fig. 8. Analysis of probed location labelled A. 10

Supplementary Note 2. Calculation of faradaic charges and capacitance values discussed in the main text. 11

Supplementary Fig. 9. Heat map of the total integrated faradaic charge. 12

Supplementary Note 3. Complementary TEY-XAS measurements on thick MXene electrodes. 13

Supplementary Fig. 10. Complementary TEY-XAS measurements on thick MXene electrodes. 13

Supplementary Fig. 11. Corresponding flake images for Fig. 3. 14

Supplementary Fig. 12. Scatter plot and corresponding flake images for cathodic cycling. 15

Supplementary Fig. 13. Complete set of SECCM voltammograms in the hydrogen evolution reaction (HER)-capacitive region 16

Supplementary Fig. 14. Electrochemical response of carbon and MXene flakes. 17
Supplementary Fig. 15. Set of SECCM voltammograms in the HER-capacitive region for three different grids. 18
Supplementary Fig. 16. Analysis of capacitive and anodic peak charges in the HER-capacitive region. 19
Supplementary Fig. 17. Capacitance heat maps. 20
Supplementary Fig. 18. Additional data relative to Fig. 4. 21
Supplementary Fig. 19. Bulk electrochemistry of MXene electrode. 22
Supplementary Fig. 20. Distribution of anodic peak potential. 23
Supplementary Fig. 21. Additional data to support Fig. 4. 24
Supplementary Fig. 22. Additional data relative to Fig. 5. 25
Supplementary Fig. 23. XRD pattern and SEM following synthesis. 26

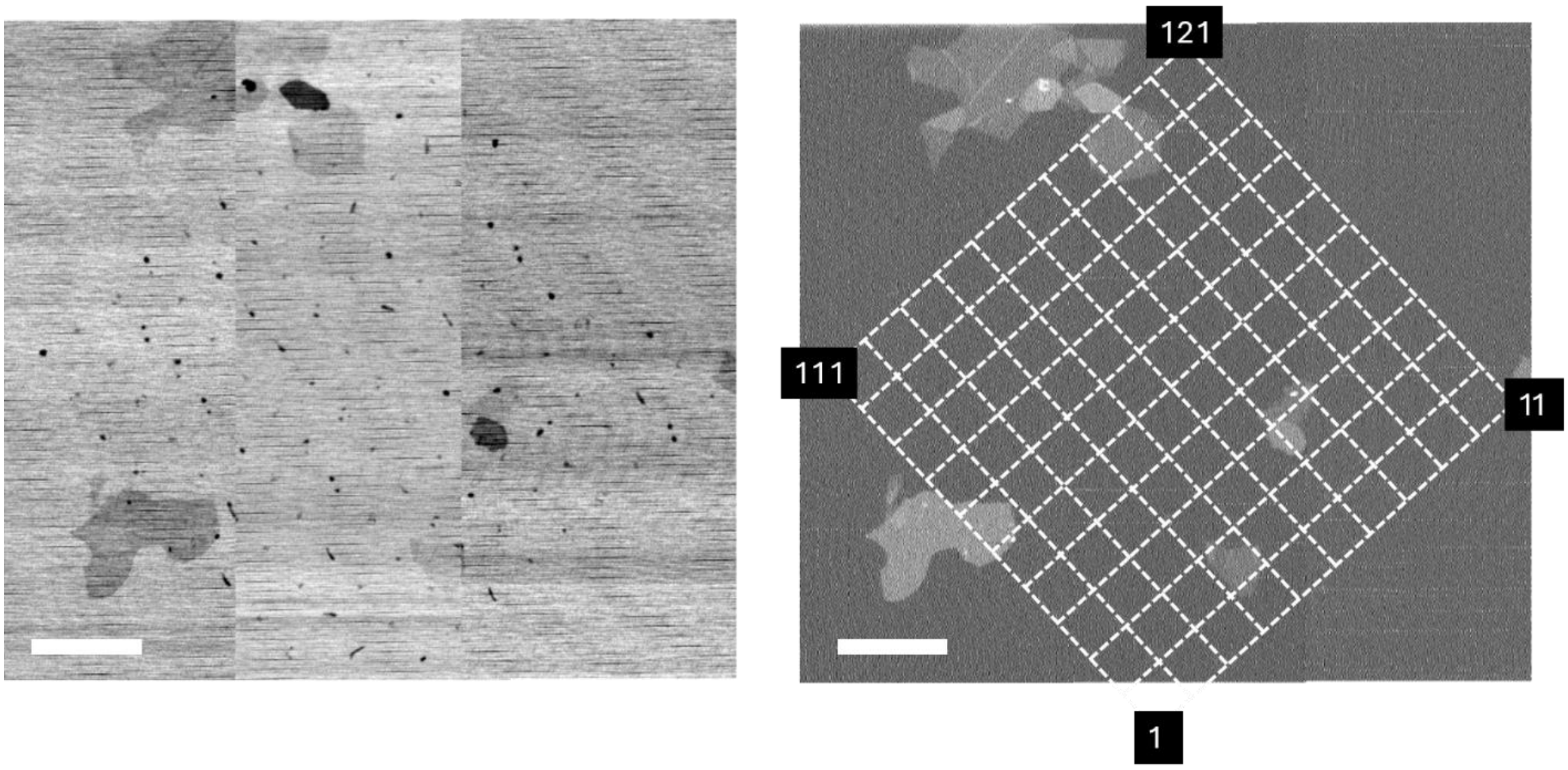

Supplementary Fig. 1. Composite scanning X-ray microscopy (SXM) images obtained at the O K-edge. Transmission (left) and total electron yield (TEY, right) images at 530 eV for the sample in Fig. 2; the entire region probed via scanning electrochemical cell microscopy (SECCM) is shown after stitching of images with smaller fields of view. The electrolyte residues resulting from SECCM measurements over an 11×11 grid of points are visible in transmission contrast. The TEY image includes added dashed lines to evidence grid point positions in relation to MXene flakes, while corner points are indexed with reference to the voltammograms reported in Supplementary Fig. 2. Scale bars: 10 µm.

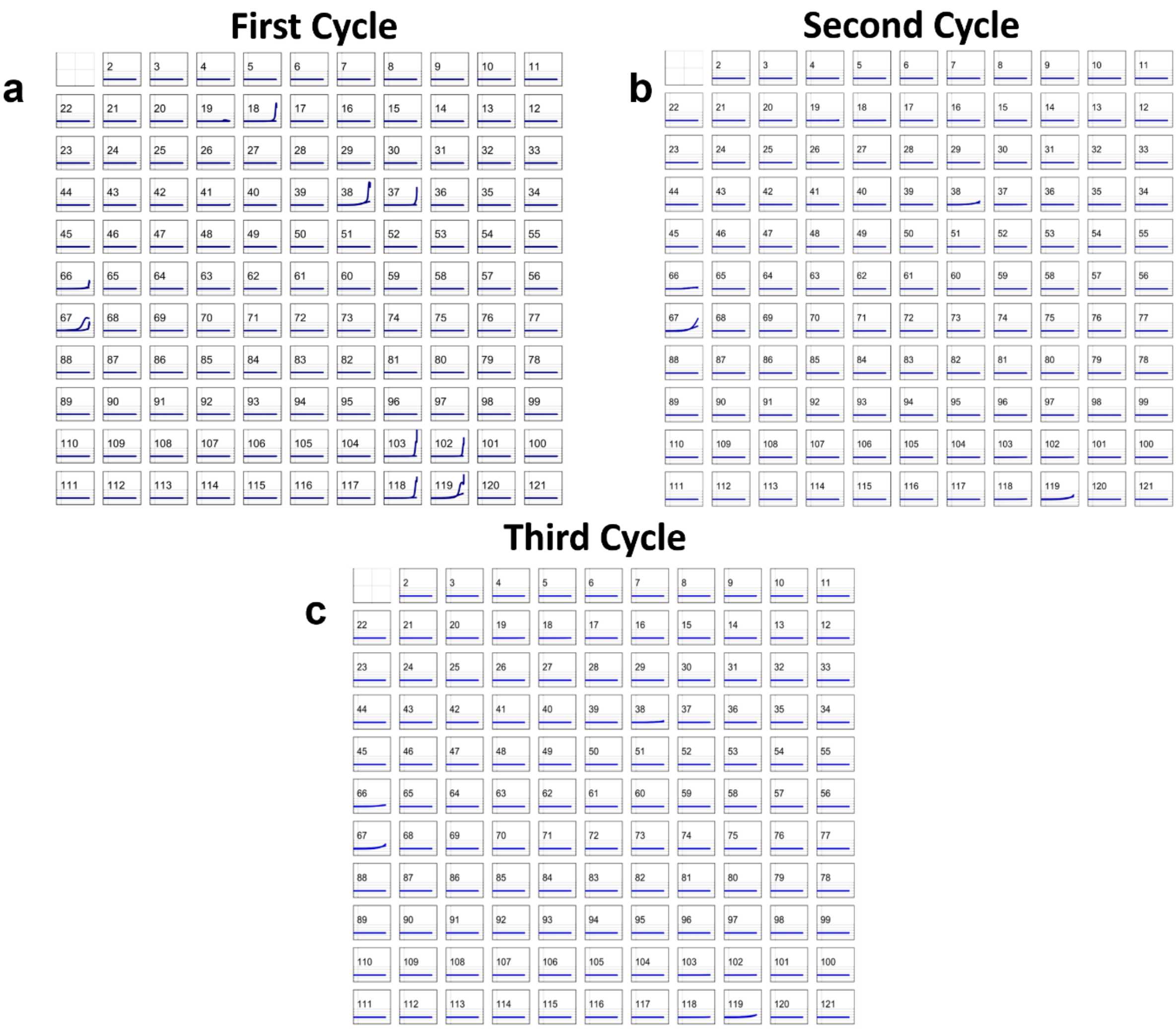

Supplementary Fig. 2. Complete set of SECCM voltammograms in the anodic region. Voltammograms obtained for the sample of MXenes drop-cast onto graphitized carbon thin film support that gave rise to images shown in Fig. 2, and Supplementary Fig. 1, 7a and 9a. The first (a), second (b) and third (c) cycles of voltammograms obtained using 10 mM H_2SO_4 at 0.5 V s^{-1} at each of the positions of the 11×11 grid are shown; points are indexed according to the order in which the locations were probed. Plots span the 0.5 – 1.3 V vs Pd-H and -0.5 to 2 pA ranges along the x- and y-axes, respectively. Points #66 and #67 and #88 correspond to those labelled as A, B and C in Fig. 2a.

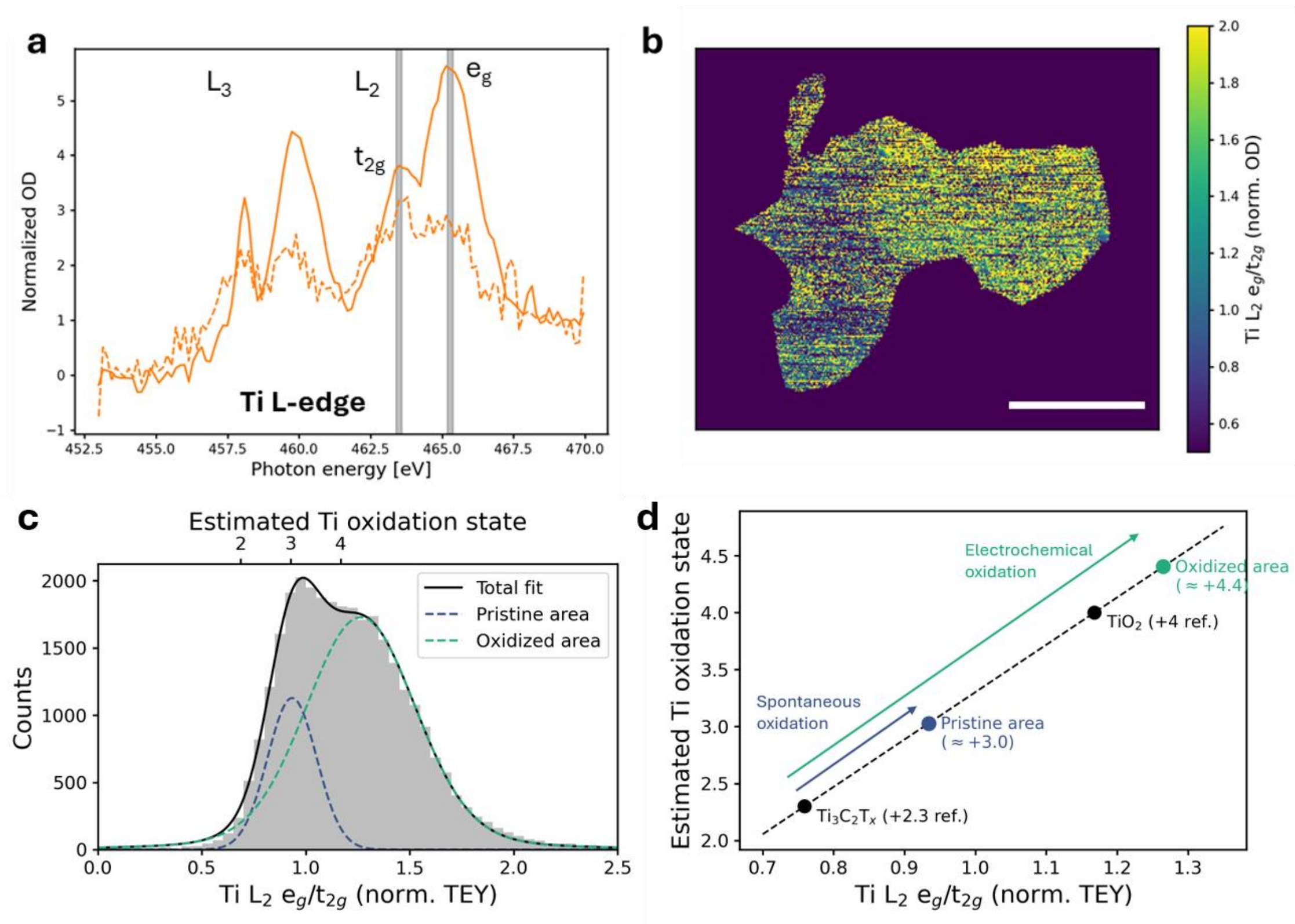

Supplementary Fig. 3. Additional data relative to Fig. 2. (a) Transmission X-ray absorption spectra (XAS) at the Ti L-edge collected in the regions highlighted in orange in Fig. 2c. (b) Ti L_2 e_g/t_{2g} peak ratio heatmap calculated from the transmission images. Scale bar: 5 µm. (c) Pixel intensity histogram of the Ti L_2 e_g/t_{2g} peak ratio heatmap calculated from the TEY images (Fig. 2e). It displays two distinct contributions (fitted with a Gaussian and a Voigt function, respectively), corresponding to the pristine and oxidized areas. The Ti oxidation state, shown as a secondary x-axis at the top of the panel, was estimated using linear interpolation between the peak ratio values of pristine $Ti_3C_2T_x$ (oxidation state +2.3) and TiO_2 (oxidation state +4.0) reference samples. The corresponding spectra are shown in Supplementary Fig. 10a. The calibration curve is presented in (d), along with the data points corresponding to the pristine and oxidized areas of Fig. 2e. Source data for the graphs presented in (a), (c) and (d) are provided in the Source Data file.

Supplementary Note 1. Optical density of a monolayer of $Ti_3C_2T_x$.

To determine the optical density (*OD*) of a monolayer of $Ti_3C_2T_x$ directly from STXM images, we analysed three images (Supplementary Fig. 3a-c) displaying a wide range of flake thicknesses and fitted their combined *OD* histograms with a Gaussian comb (Supplementary Fig. 3d-e).

A Gaussian comb is a sum of N Gaussian functions (here with the same width, σ), whose centre positions are separated by a constant interval, Δ. Assuming that *OD* scales linearly with the number of layers—which is valid for *OD* values below 1—even if a true monolayer is not captured in these images, the separation Δ between two consecutive distributions can still be used to estimate the *OD* of a single layer. Using this approach, we estimate the *OD* of a $Ti_3C_2T_x$ monolayer to be 0.016.

It should be noted that in the main text (e.g., in Fig. 2f), the *OD* was normalized to the pre-edge signal (ΔOD) to remove contributions from electrolyte residues, whereas in this analysis, the raw *OD* values were used for fitting. This might lead to a slight overestimation of Δ. However, for pristine MXenes, the pre-edge absorption is very small compared to that at 463 eV, and we still obtain an excellent match between the average *OD* values in Fig. 2f and 4c and the Δ determined here.

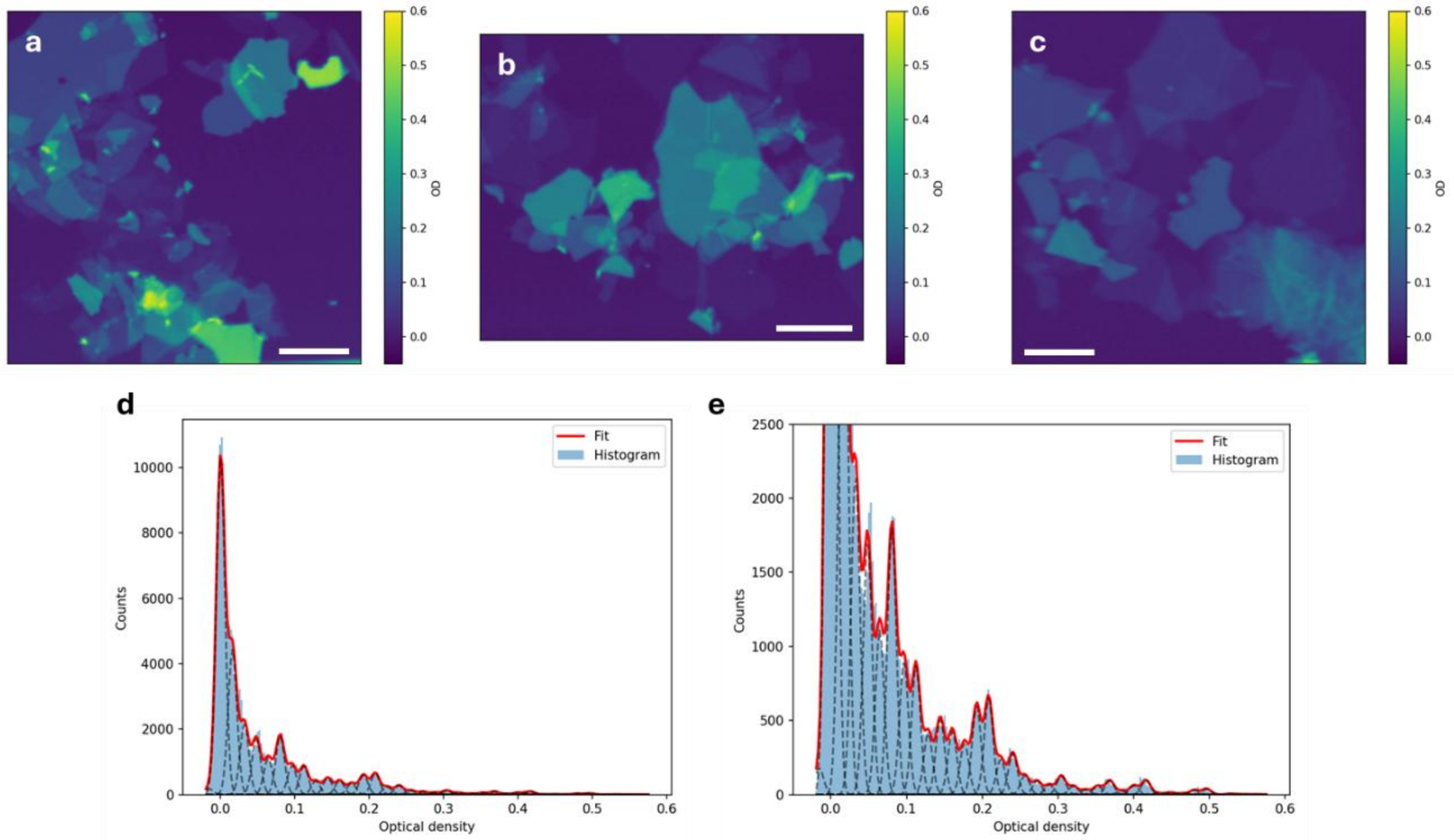

Supplementary Fig. 4. Optical density of a monolayer of $Ti_3C_2T_x$. (a–c) STXM images of pristine $Ti_3C_2T_x$ MXene flakes acquired at 463 eV. (d) Gaussian comb fit of the merged *OD* histograms obtained from (a–c). Fitting parameters: $N = 42$, $\sigma = 0.006$, $\Delta = 0.016$, offset = 0.001. (e) Zoomed-in view highlighting the quality of the fit at higher *OD* values. Scale bars: 5 µm. Source data for the graphs presented in (d) and (e) are provided in the Source Data file.

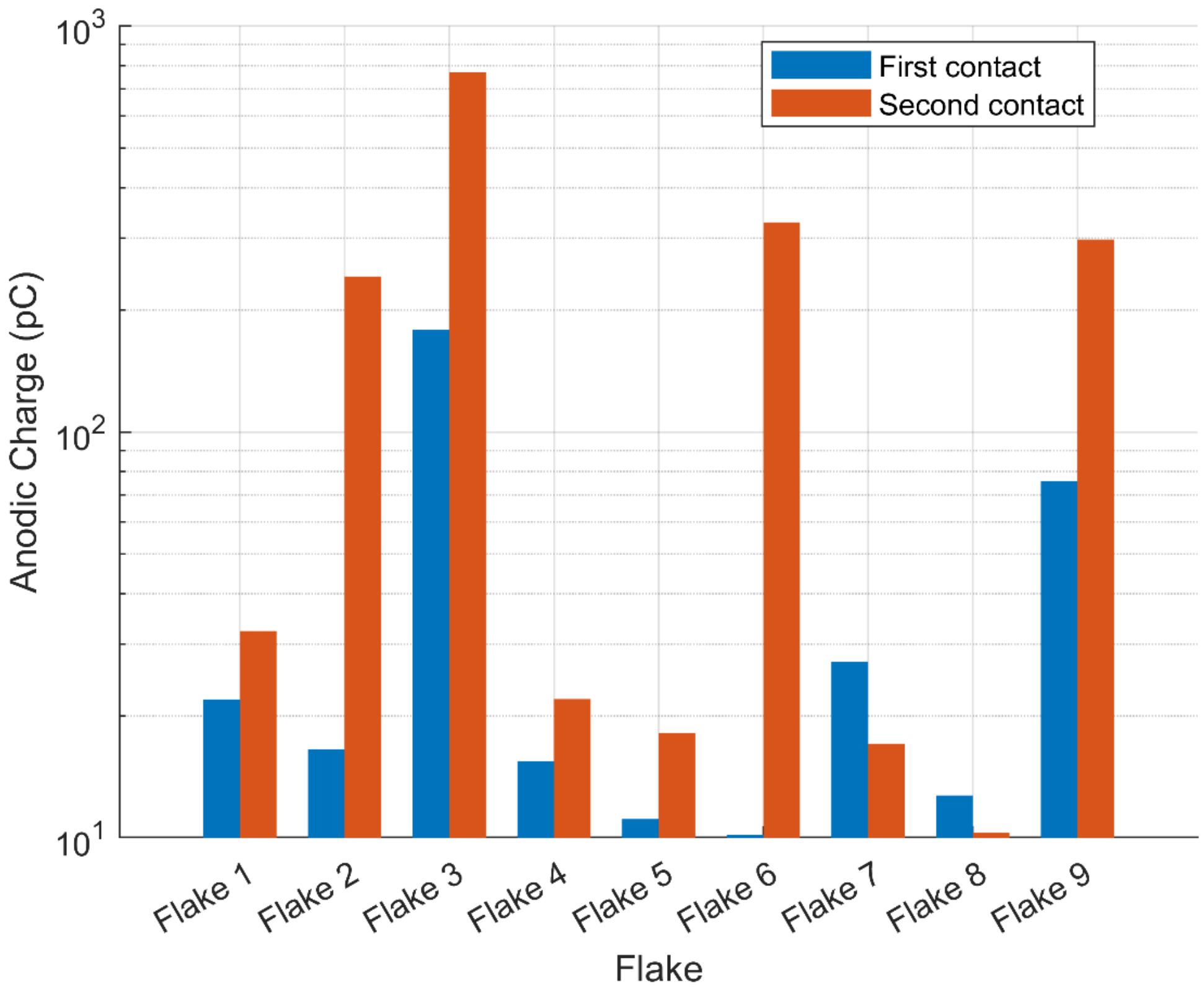

Supplementary Fig. 5. Priming effect. Faradaic charge obtained at each of the nine flakes that were probed with the nanopipette on more than one location, as shown in Supplementary Fig. 9. The chart shows the total charge measured at the first two consecutive contacts of these flakes, calculated using methods detailed in Supplementary Fig. 8. Source data are provided in the Source Data file.

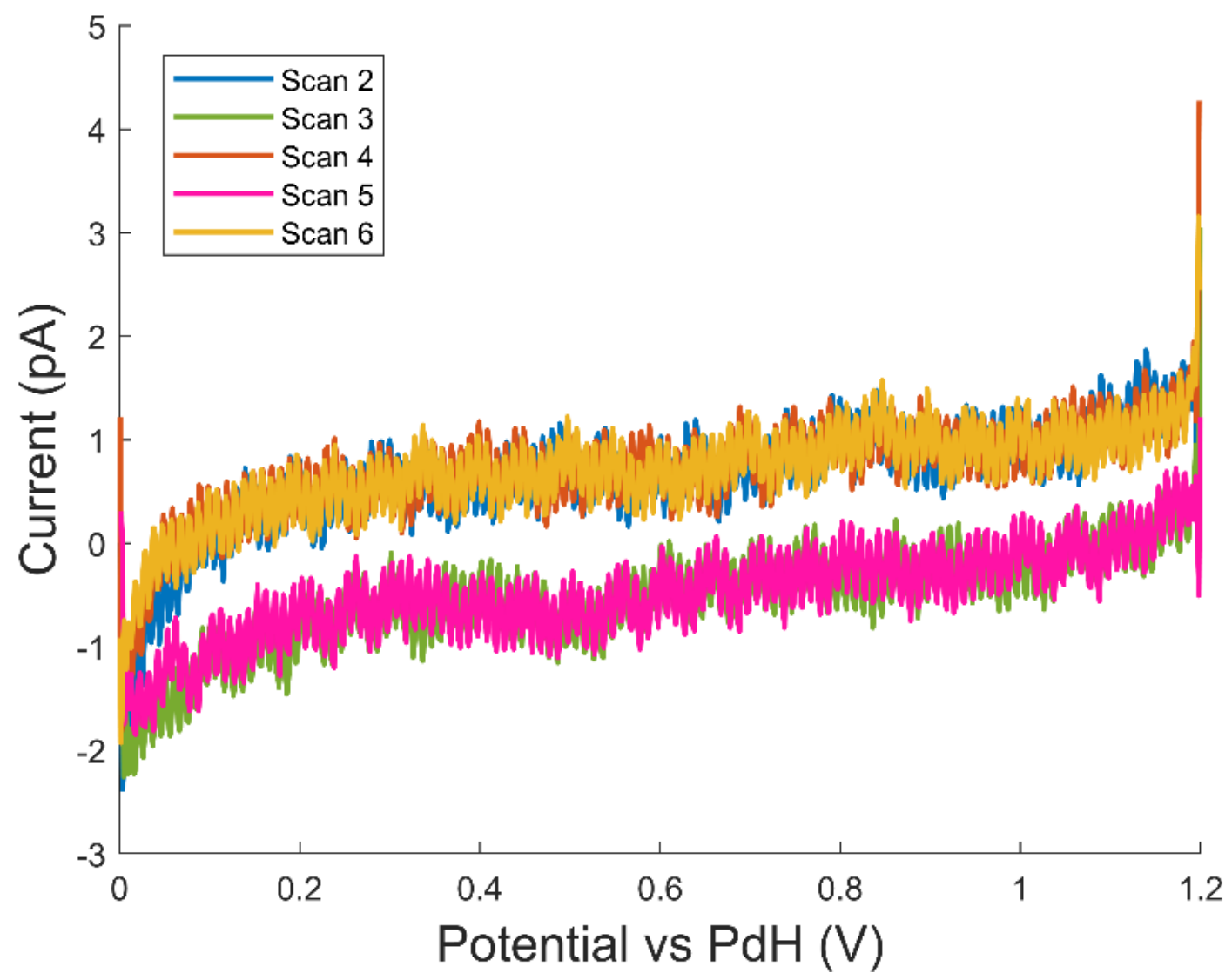

Supplementary Fig. 6. Expanded plot of anodic and cathodic potential sweeps at carbon. Plot of all anodic and cathodic potential sweeps obtained at point #88 (Supplementary Fig. 2) corresponding to the probed area in contact with the graphitized carbon thin film electrode at the location marked as C in Fig. 2a. A total of 6 sweeps were obtained and sweeps 2-6 are shown numbered in the order of measurement. The first sweep at each point is obtained during the approach of the nanopipette probe and is therefore not used for analysis of the current response. The sweeps show overlapping currents of ca. 1 pA and the characteristic response of double layer capacitance, as discussed in the main text. Source data are provided in the Source Data file.

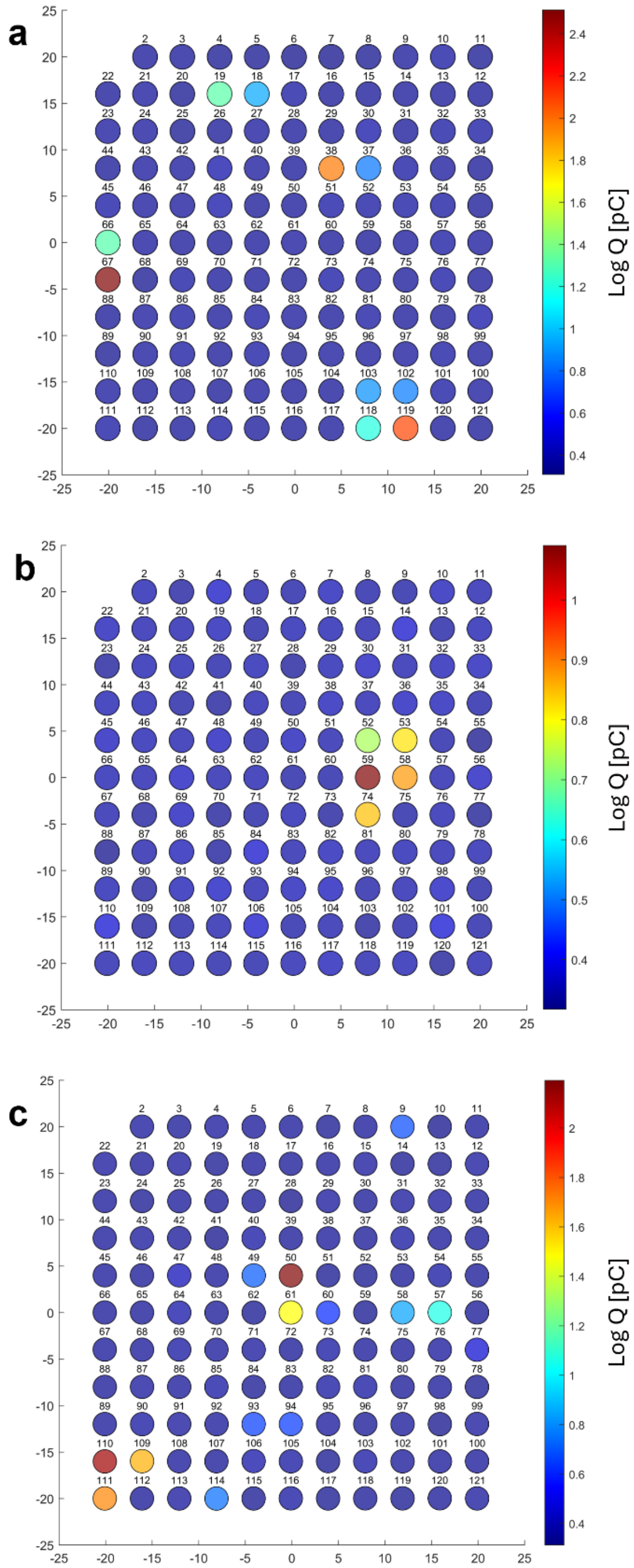

Supplementary Fig. 7. Heat map of the integrated charge of the first anodic sweep. (a) shows the grid discussed in Fig. 2, and Supplementary Fig. 1, 2 and 9a and (b-c) two additional 11×11 grids characterized via SECCM using the same nanopipette probe and identical conditions. The integrated charges are uniformly small at points in contact with the graphitized thin film substrate (ca. 2 pC) but show higher values in correspondence with contacted sub-domains of MXene flakes.

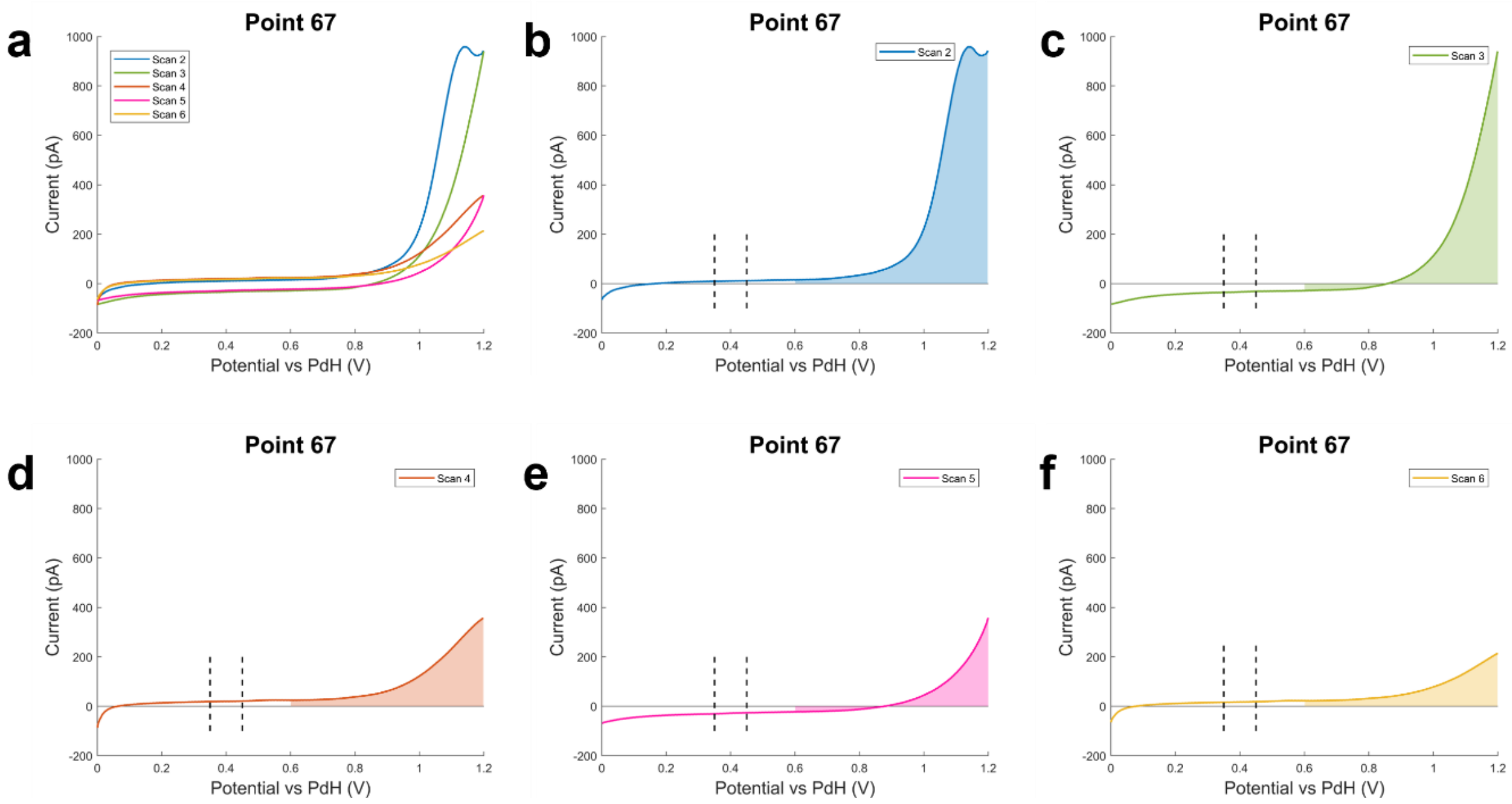

Supplementary Fig. 8. Analysis of probed location labelled A. (a) Current sweeps obtained at point #67 (Supplementary Fig. 2), corresponding to the probe making contact with a MXene flake at the location labelled A in Fig. 2a. Sweeps 2-6 are shown numbered in the order of measurement; the first sweep at each point is obtained during the approach of the nanopipette probe and is therefore not used for analysis of the anodic charge. (b-f) Illustration of the integration procedure adopted to estimate the total anodic charge passed at the MXene sub-domain. For each sweep the total integrated charge was calculated over the interval 0.6 to 1.2 V and these values were corrected for capacitive contributions by assuming a constant capacitive current equal to the mean current in the 0.35 to 0.45 V window (dashed lines). A similar process was used for the analysis of all points in the SECCM grid, yielding the heat map shown in Supplementary Fig. 9. Source data are provided in the Source Data file.

Supplementary Note 2. Calculation of faradaic charges and capacitance values discussed in the main text.

Considering a lattice parameter a = 3.03 Å for the hexagonal cell of $Ti_3C_2T_x$,[1] it is possible to calculate the area defined by a unit cell (one side). The total faradaic charge Q passed for a process involving n electrons/unit cell, over an area A of a MXene monolayer flake can then be calculated as:

$$Q = \frac{nQ_eA}{A_{cell}} \quad (1)$$

where Q_e is the electron charge. Oxidized area estimates reported in the text were calculated from this same relationship, but using the charge as input value.

Several products have been considered possible for the oxidation of MXenes;[2,3] depending on the final product of the oxidation, it is possible to write the following net reactions with maximum n_{max} = 12, 16 or 20:

$$\mathrm{Ti_3C_2T_x + 6H_2O \longrightarrow 3TiO_2 + 2C + 12H^+ + (12 - x)e + xT^-} \quad (2)$$

$$\mathrm{Ti_3C_2T_x + 8H_2O \longrightarrow 3TiO_2 + 2CO + 16H^+ + (16 - x)e + xT^-} \quad (3)$$

$$\mathrm{Ti_3C_2T_x + 10H_2O \longrightarrow 3TiO_2 + 2CO_2 + 20H^+ + (20 - x)e + xT^-} \quad (4)$$

Given that the area defined by the nanopipette aperture is *ca.* 0.5 μm^2, the maximum anodic charge expected for an anodic process limited to a monolayer area of this size is 19.8 pC, and is obtained by combining Supplementary Equation (1) and (4).

The gravimetric specific capacitance C_s was calculated from the total capacitance C using the following expression:

$$C_s = \frac{C\,SSA_{1side}(1ML)}{A_{flake}} \quad (5)$$

where A_{flake} is the total geometric area of the flake in contact with the nanopipette probe and $SSA_{1side}(1ML) = 272\ \mathrm{m^2g^{-1}}$, is the specific surface area of 1 side of a MXene monolayer.[4]

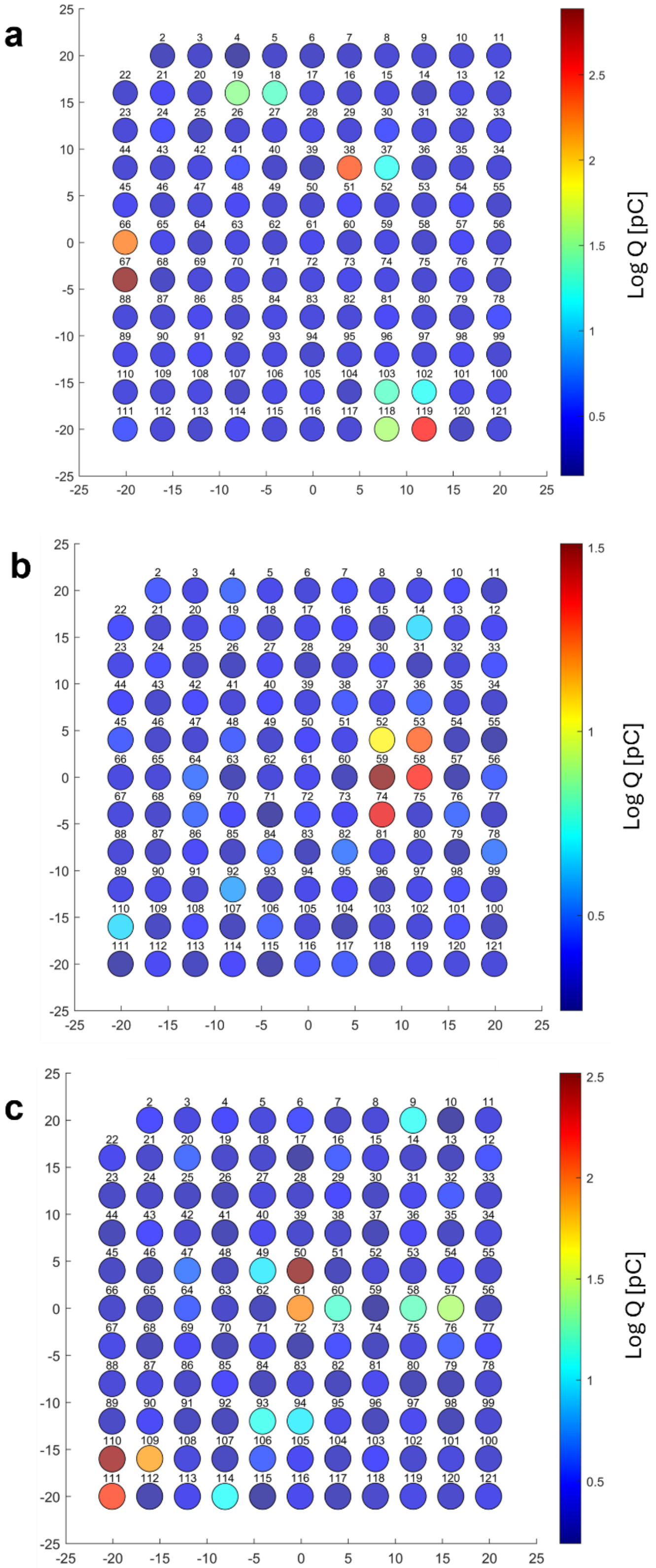

Supplementary Fig. 9. Heat map of the total integrated faradaic charge. Heat maps calculated over all cycles for (a) the grid discussed in Fig. 2, and Supplementary Fig. 1, 2 and 7a and (b-c) two additional 11×11 grids characterized via SECCM using the same nanopipette probe and identical conditions. The charges measured at points in contact with the graphitized thin film substrate yielded *ca.* zero charge as expected after successful capacitive correction.

Supplementary Note 3. Complementary TEY-XAS measurements on thick MXene electrodes.

To gain further insight into the oxidation mechanism probed in our anodic cycling experiments, complementary TEY-XAS measurements were performed at the HE-SGM beamline of BESSY II, focusing in particular on the C K-edge. The C K-edge provides valuable information on the structure and surface chemistry of MXenes,[5] but it is challenging to probe in STXM due to geometrical constraints. In the present case, these measurements were not feasible anyway because of the strong background signal from the carbon coating.

Therefore, C K-edge measurements were conducted on a separate beamline dedicated to soft X-ray absorption at the C K-edge, using micrometre thick free-standing MXene films prepared by vacuum filtration. Electrochemical oxidation of these films was carried out in a conventional three-electrode cell, with an Ag/AgCl reference electrode and a carbon counter electrode, cycling several times over the 0.2-1 V window at 10 mV s^{-1} in 0.1 M H_2SO_4.

As shown in Supplementary Fig. 10a, the Ti L-edge spectra indicate an increase in the Ti oxidation state, consistent with our STXM observations. At the C K-edge (Supplementary Fig. 10b), the MXene spectral features disappear entirely after oxidation. The residual signal could correspond to amorphous carbon formation, although further investigation would be required to confirm this and is beyond the scope of the present study.

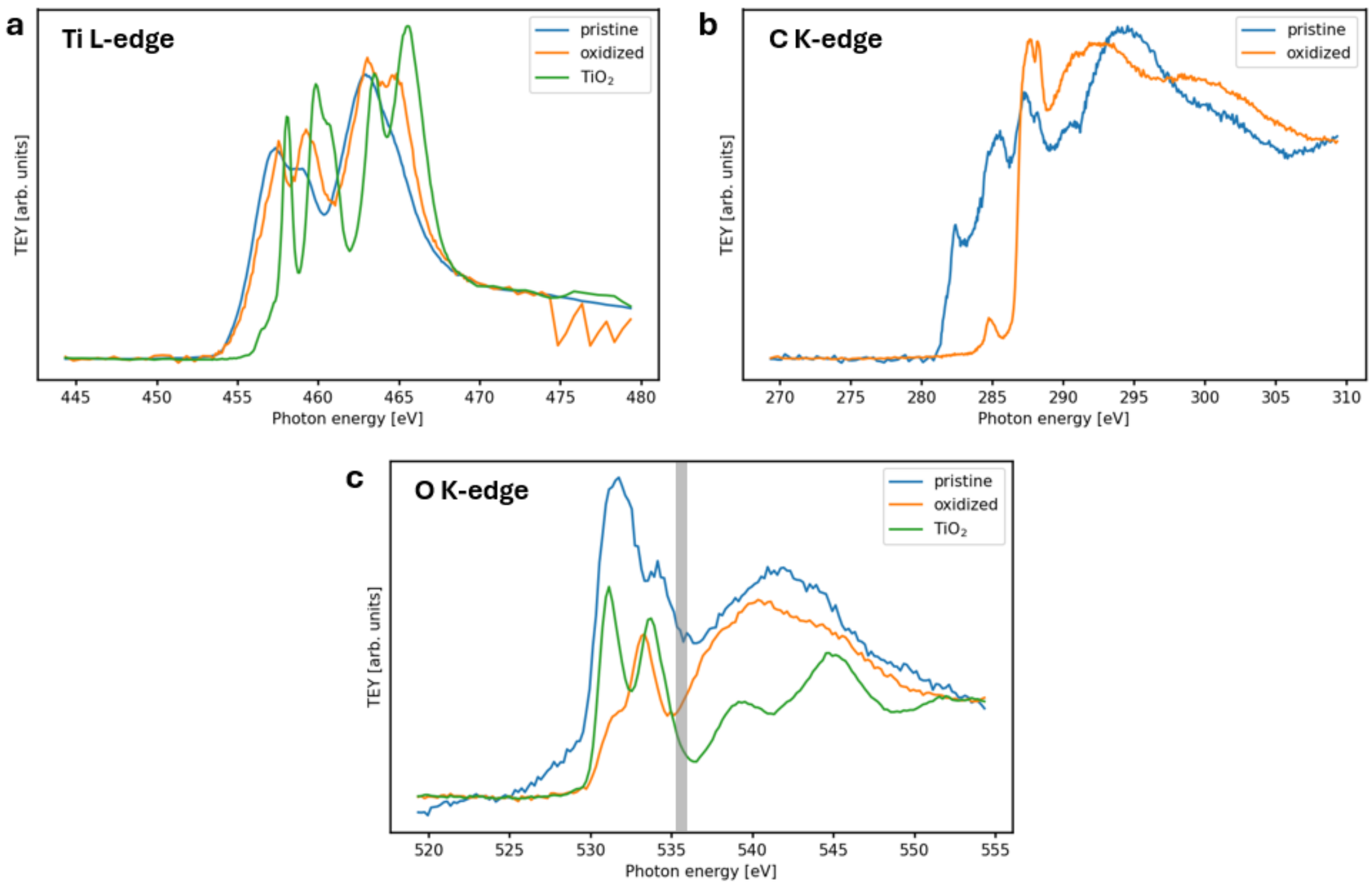

Supplementary Fig. 10. Complementary TEY-XAS measurements on thick MXene electrodes. (a) Ti L-edge spectra of a pristine MXene film (blue), the same film after electrochemical oxidation (orange), and a TiO_2 powder reference (green). (b) C K-edge spectra of the pristine (blue) and oxidized (orange) MXene films. (c) O K-edge spectra of a pristine MXene film (blue), the same film after electrochemical oxidation (orange), and a TiO_2 powder reference (green). Source data are provided in the Source Data file.

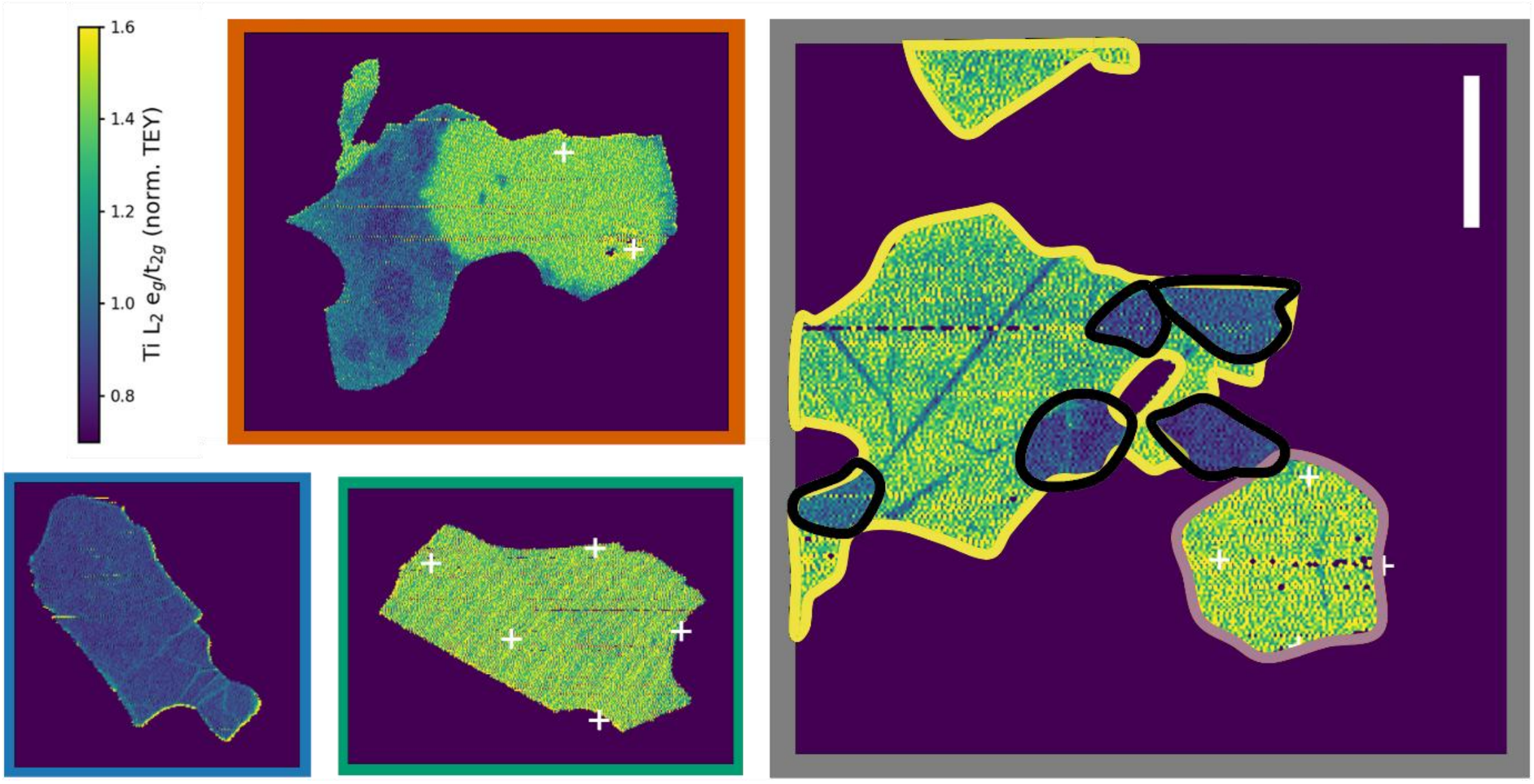

Supplementary Fig. 11. Corresponding flake images for Fig. 3. Ti L_2 e_g/t_{2g} peak ratio heatmaps of the flakes analysed to construct Fig. 3 (anodic cycling). The colour of each image's or flake's frame corresponds to the colour of the corresponding points in Fig. 3. All images are at scale; scale bar: 5 µm.

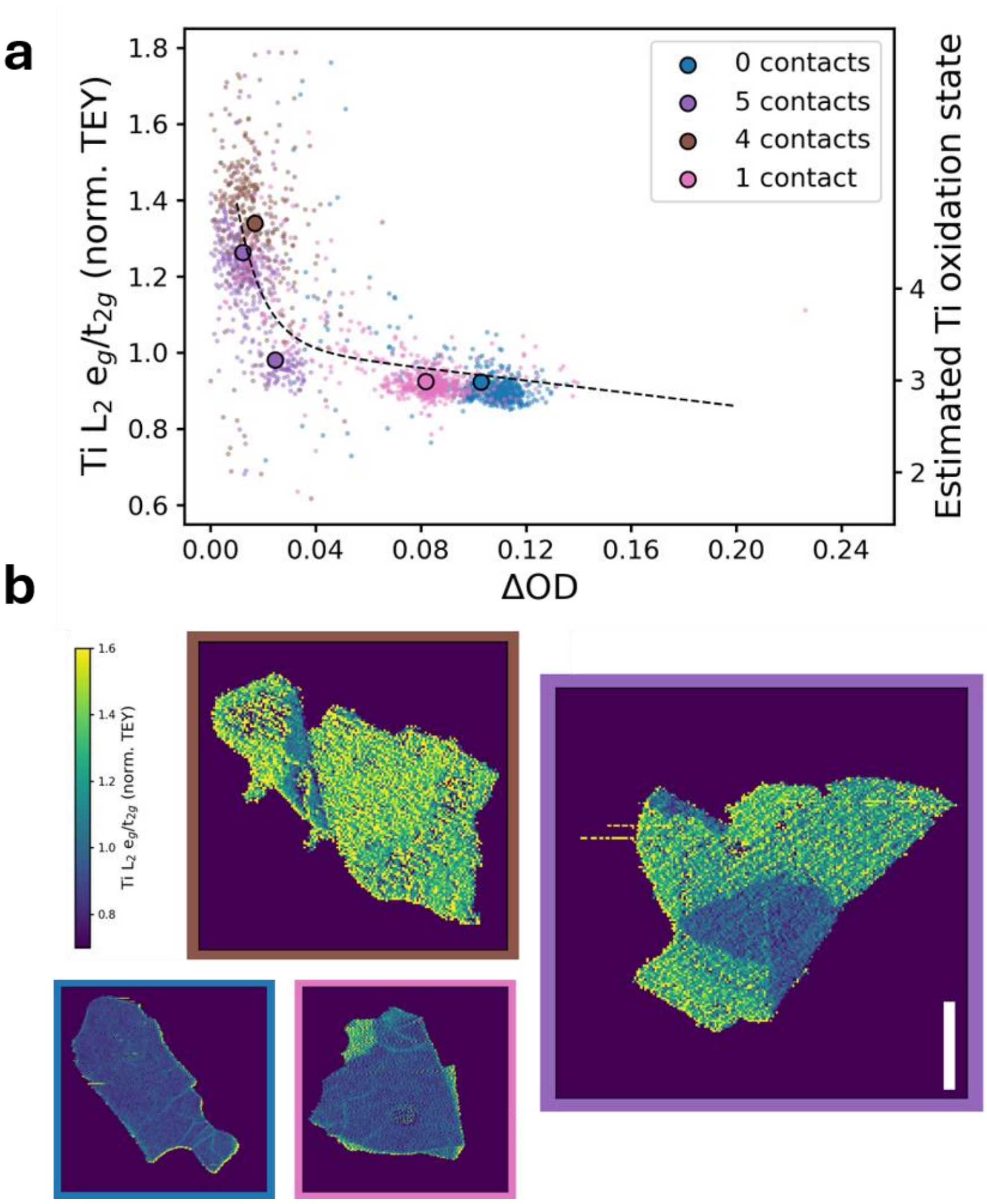

Supplementary Fig. 12. Scatter plot and corresponding flake images for cathodic cycling. (a) Pixel-by-pixel scatter plot of Ti L_2 e_g/t_{2g} ratio vs *ΔOD* for flakes cycled under cathodic conditions. The larger dots correspond to each distribution's centre of mass. (b) Corresponding flake images. The colour of each image's frame corresponds to the colour of the corresponding points in (a). All images are at scale; scale bar: 5 µm. Source data for the graphs presented in (a) are provided in the Source Data file.

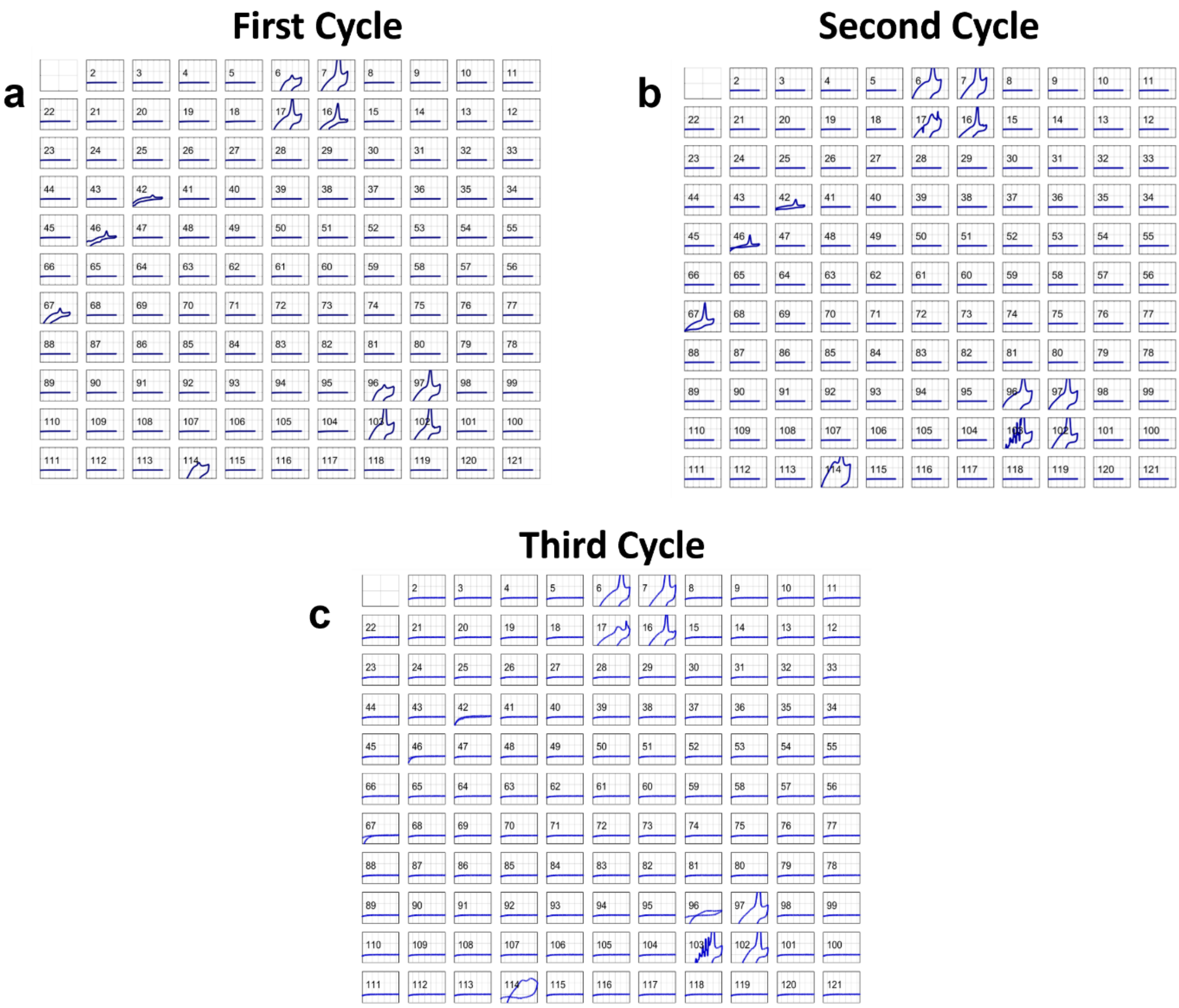

Supplementary Fig. 13. Complete set of SECCM voltammograms in the hydrogen evolution reaction (HER)-capacitive region. Voltammograms obtained from the sample of MXenes drop-cast on graphitized carbon thin film support that gives rise to images in Fig. 4 and 5. The first (a), second (b) and third (c) cycles of voltammograms obtained using 10 mM H_2SO_4 at 0.5 V s^{-1} at each of the positions of the 11×11 grid are shown; points are indexed according to the order in which the locations were probed. Plots span the -0.75 to 0.5 V vs Pd-H and -150 to 400 pA ranges along the x- and y-axes, respectively. Point #7 corresponds to voltammograms discussed in Fig. 4. Points #46 and #67 correspond to voltammograms for the two flakes discussed in Fig. 5.

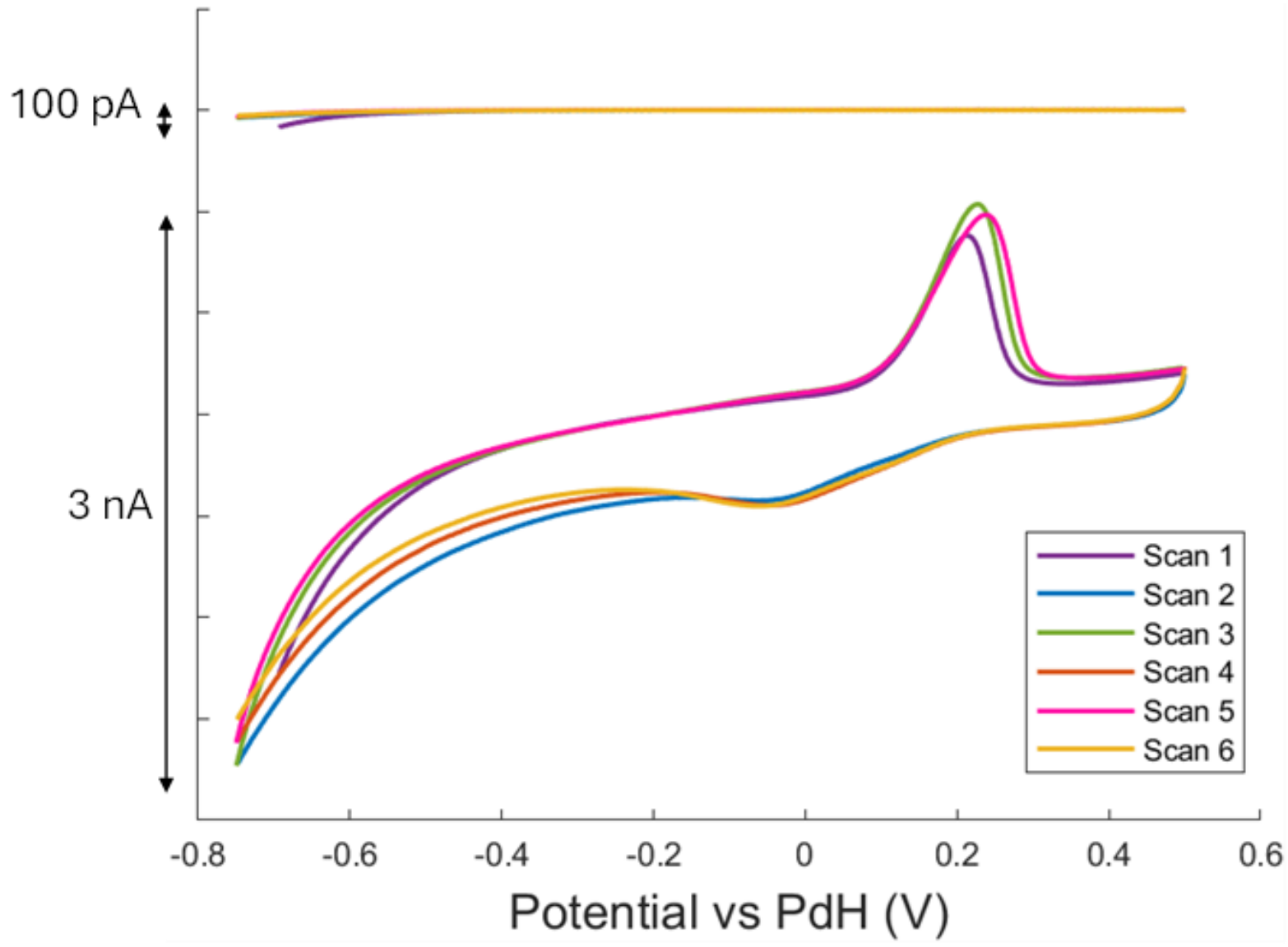

Supplementary Fig. 14. Electrochemical response of carbon and MXene flakes. Comparison of the electrochemical response in the HER-capacitive region obtained at carbon (top traces) and at MXene (bottom traces) contacted sub-domains. MXene response comes from point #7 in Supplementary Fig. 13. Source data are provided in the Source Data file.

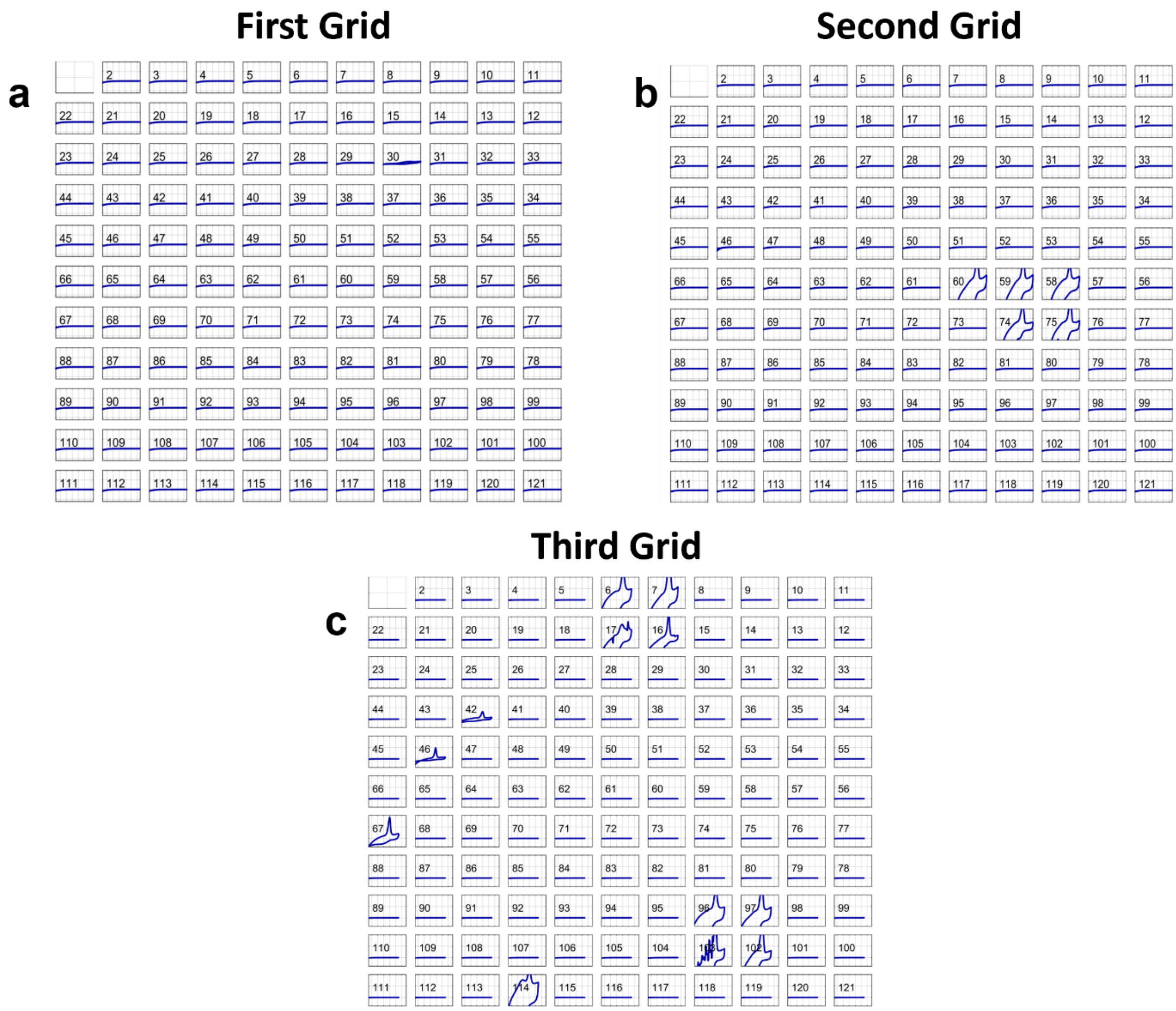

Supplementary Fig. 15. Set of SECCM voltammograms in the HER-capacitive region for three different grids. SECCM voltammograms of the 2nd cycle obtained in the HER-capacitive region for three different SECCM grids: (a-b) show two 11×11 grids characterized via SECCM using the same nanopipette probe and identical conditions as for the grid in (c), which displays the flakes that are subject of discussion in Fig. 4 and 5 as well as Supplementary Fig. 13 and 14. Plots span the -0.75 to 0.5 V vs Pd-H and 150 to 400 pA ranges along the x- and y-axes, respectively.

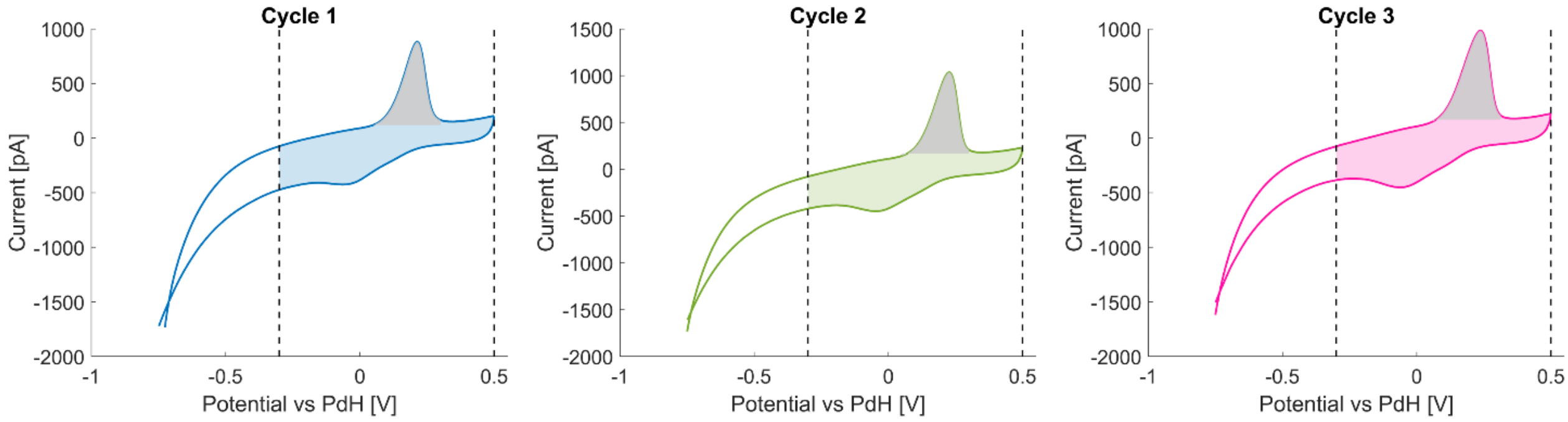

Supplementary Fig. 16. Analysis of capacitive and anodic peak charges in the HER-capacitive region. Above gives an illustrative example for calculating the capacitance for each cycle. For each cycle the total integrated charge was calculated over the interval 0.5 to -0.3 V. The charge anodic peak charge was calculated and taken away to yield the square charge for each cycle. These values were then divided by the potential window (1.6 V) to yield total capacitance. This process was used for the analysis of all points in the SECCM grid, yielding the heat map shown in Supplementary Fig. 17. Source data are provided in the Source Data file.

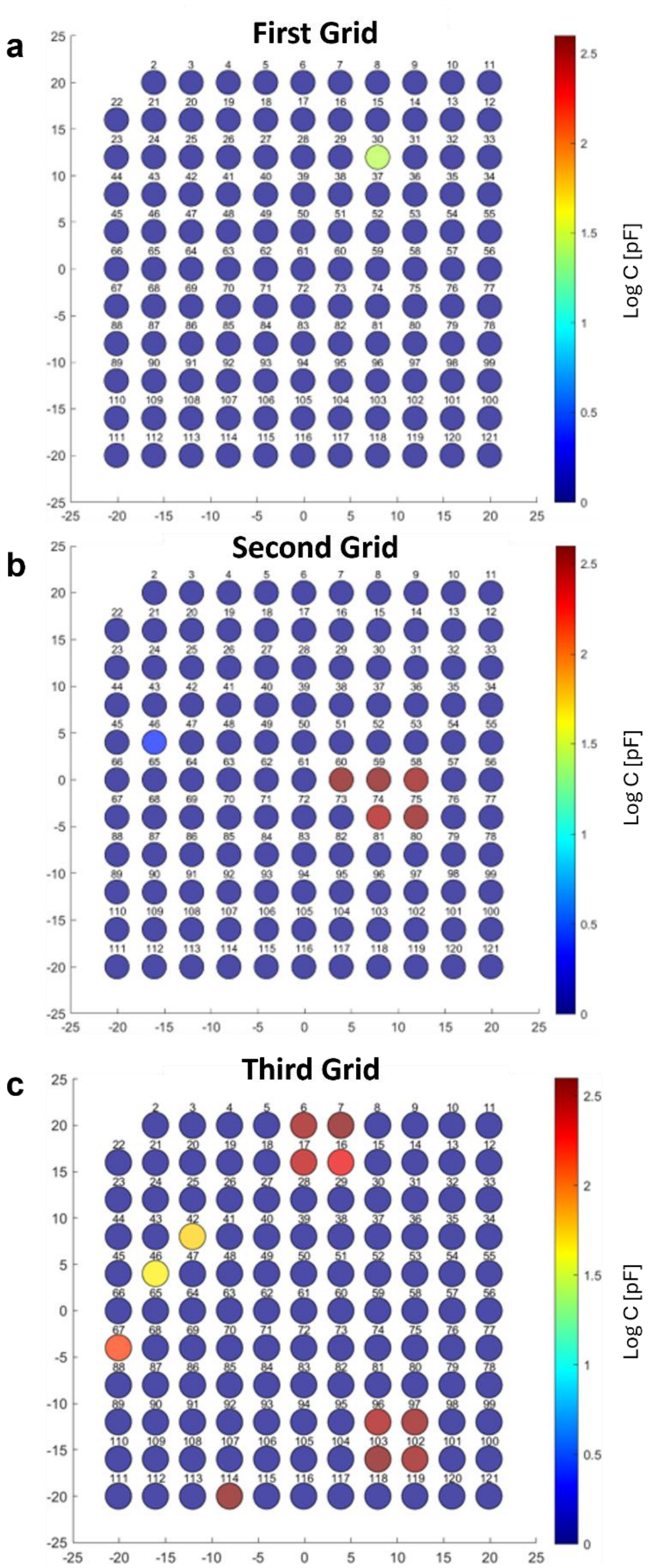

Supplementary Fig. 17. Capacitance heat maps. Heat maps of the total capacitance calculated from the 2nd cycle over the -0.3 to 0.5 V potential window for three different SECCM grids: (a-b) show two 11×11 grids characterized via SECCM using the same nanopipette probe and identical conditions as for the grid in (c), which displays the flakes that are subject of discussion in Fig. 4 and 5 as well as Supplementary Fig. 13 and 14. Note that, for most MXene sub-domains contacted, the capacitance values are 150-350 pF.

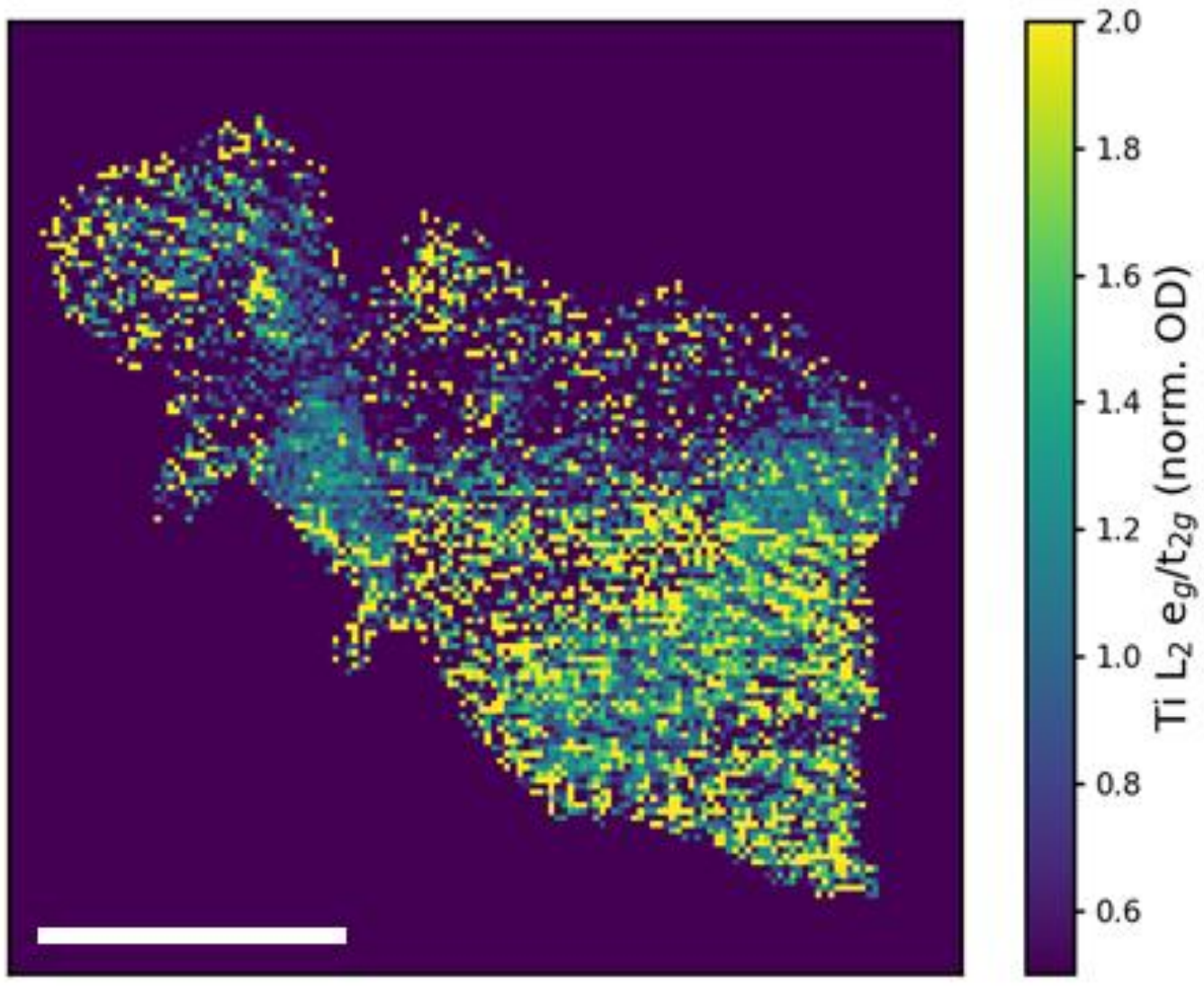

Supplementary Fig. 18. Additional data relative to Fig. 4. Ti L_2 e_g/t_{2g} peak ratio heatmap calculated from the transmission images. Scale bar: 5 µm.

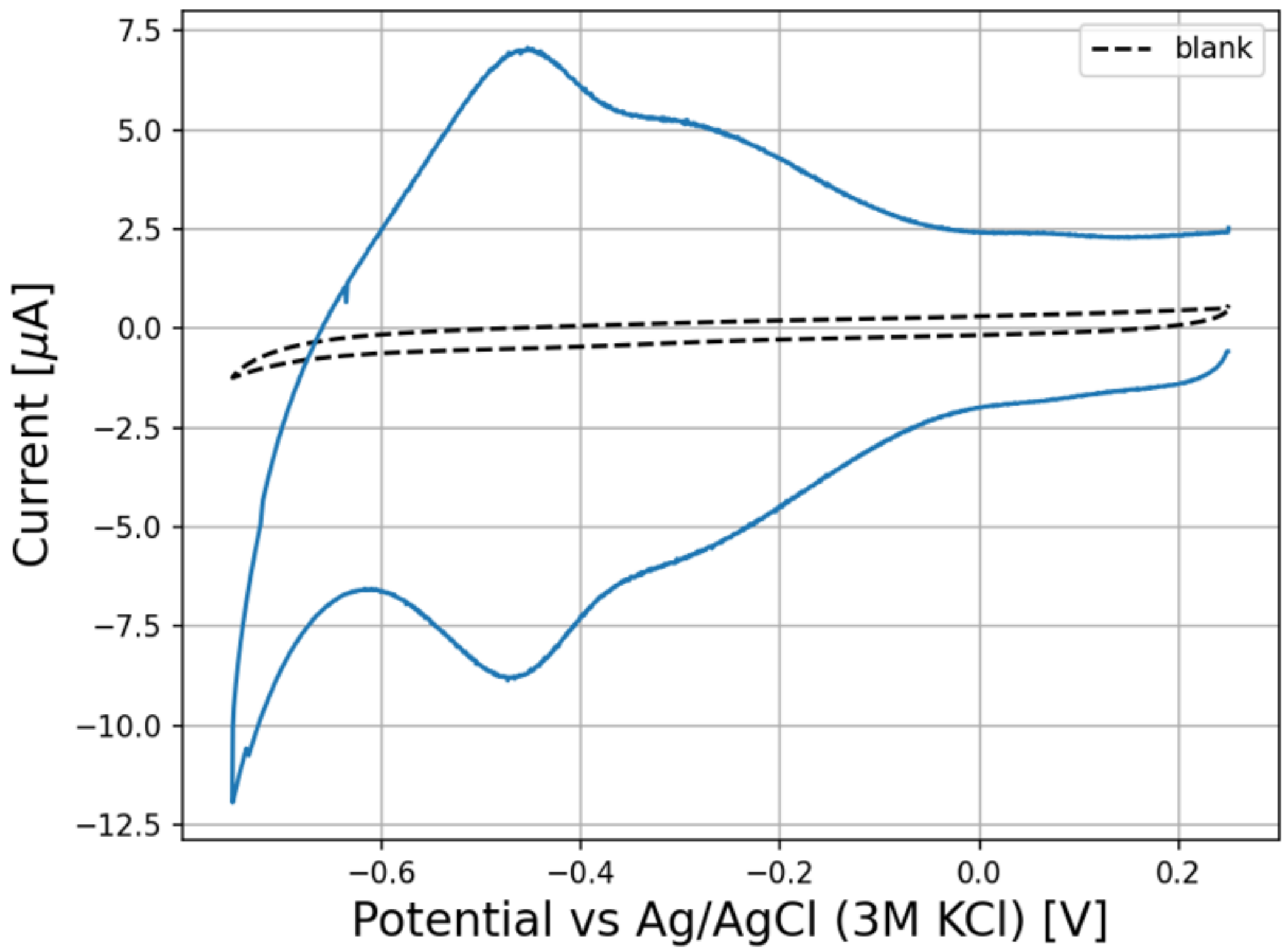

Supplementary Fig. 19. Bulk electrochemistry of MXene electrode. Cyclic voltammogram of dropcast MXene electrode in 0.1 M H_2SO_4 at 10 mV s^{-1}. Source data are provided in the Source Data file.

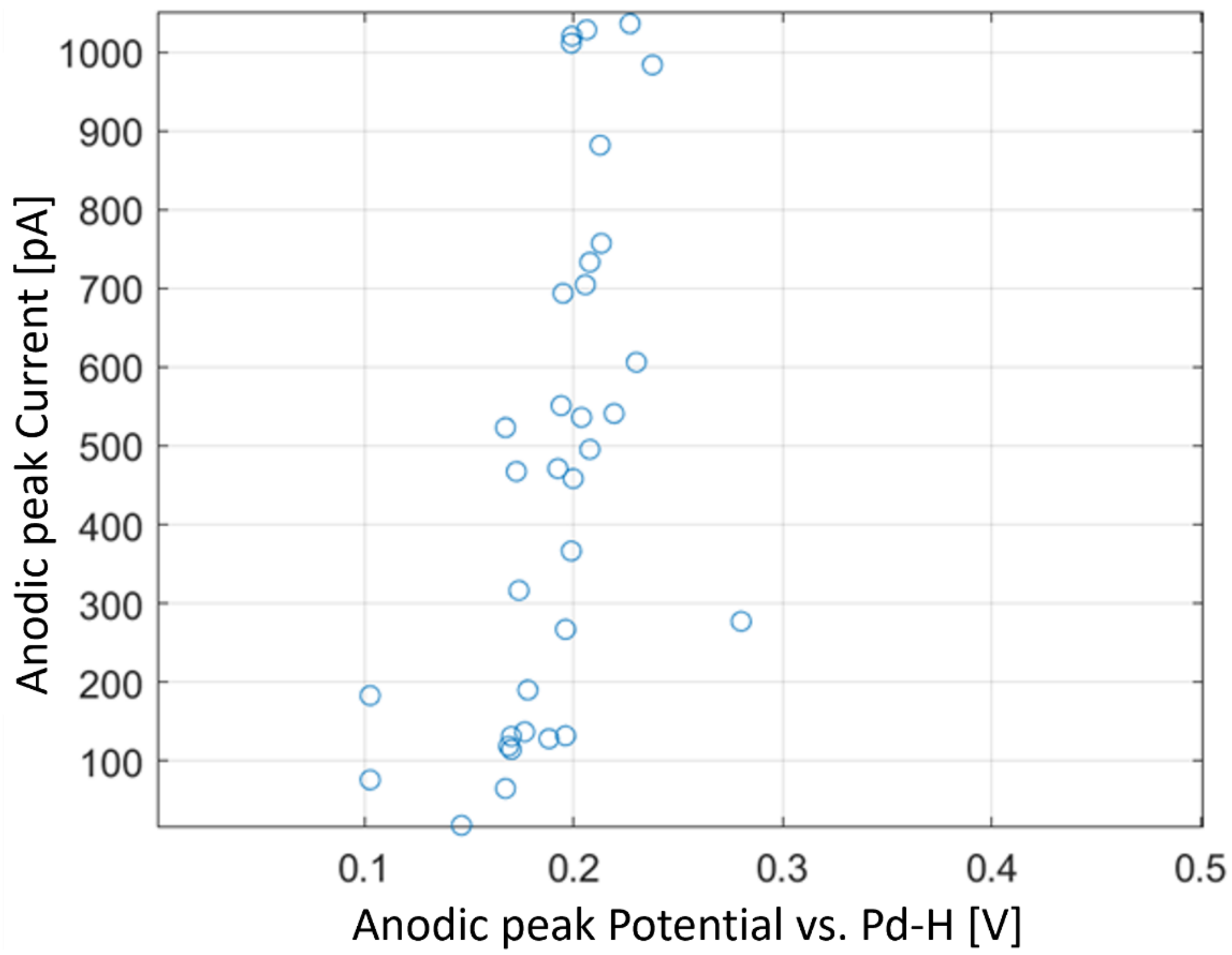

Supplementary Fig. 20. Distribution of anodic peak potential. Plot of peak current vs peak potential for peak B in anodic sweeps, discussed in Fig. 4. Source data are provided in the Source Data file.

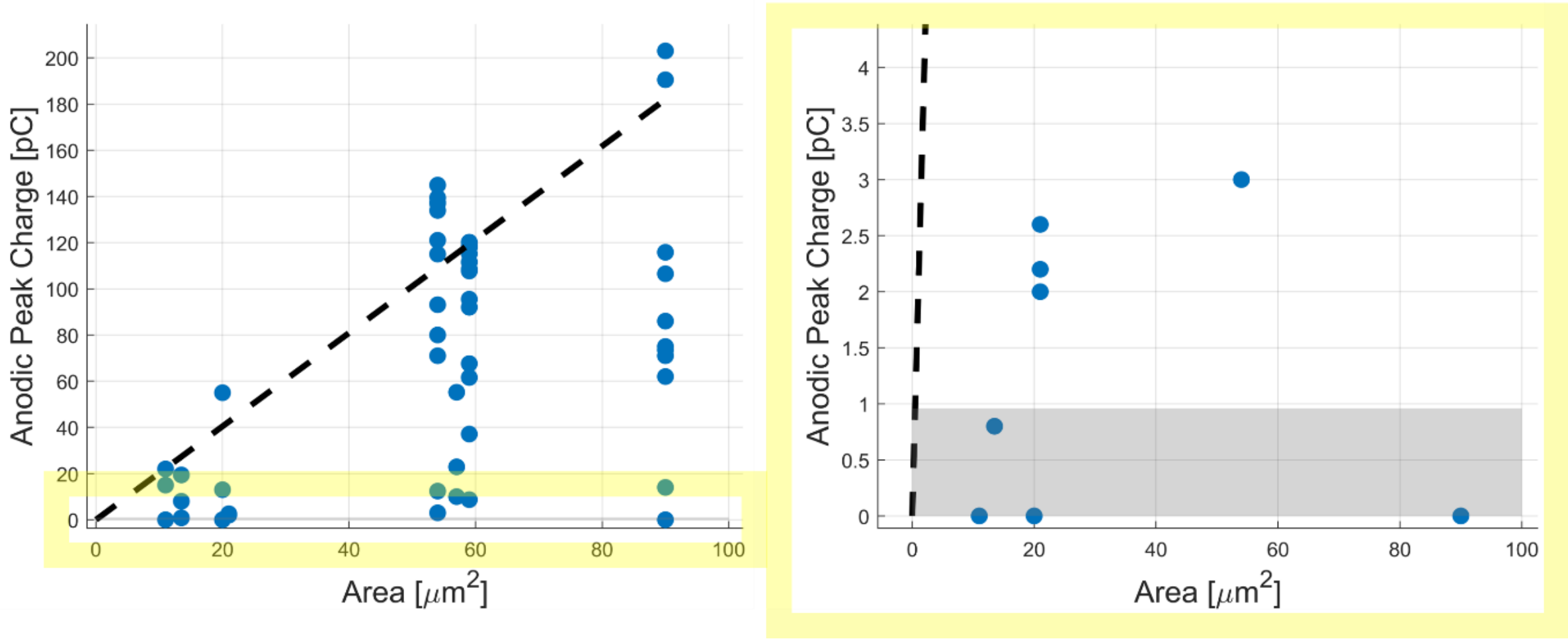

Supplementary Fig. 21. Additional data to support Fig. 4. Anodic peak charges extracted from all SECCM grids. Area highlighted in grey shows expected peak charge for a 1-electron process if confined to the probed contact area of the nanopipette (~0.5 μm^2). Source data are provided in the Source Data file.

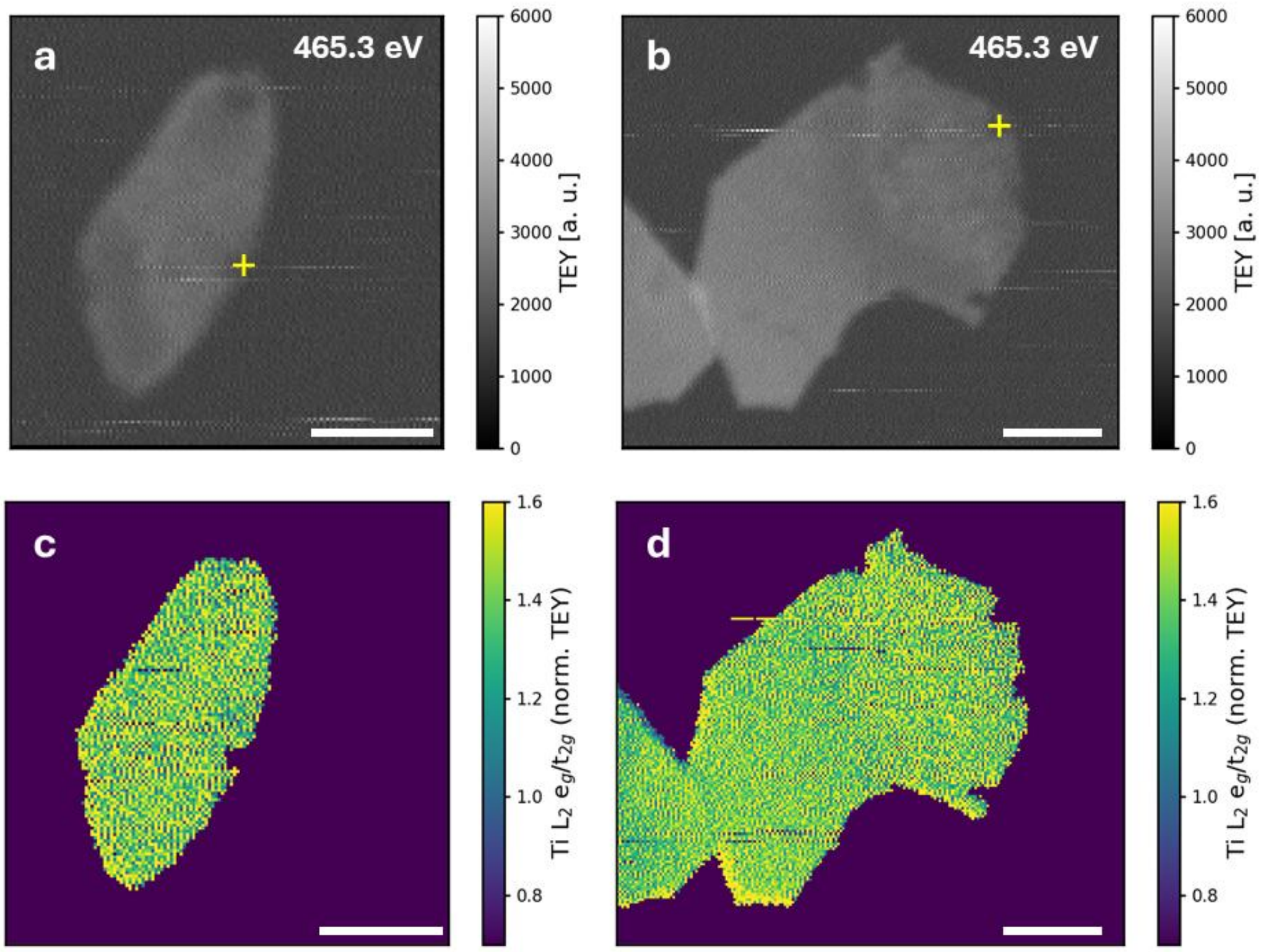

Supplementary Fig. 22. Additional data relative to Fig. 5. (a,b) TEY-SXM images acquired at 463.5 eV (Ti L-edge) in the two regions. SECCM contact points on the flakes are marked with a cross. (c,d) Corresponding Ti L_2 e_g/t_{2g} peak ratio heatmap calculated from the normalized TEY images. Scale bars: 2 µm.

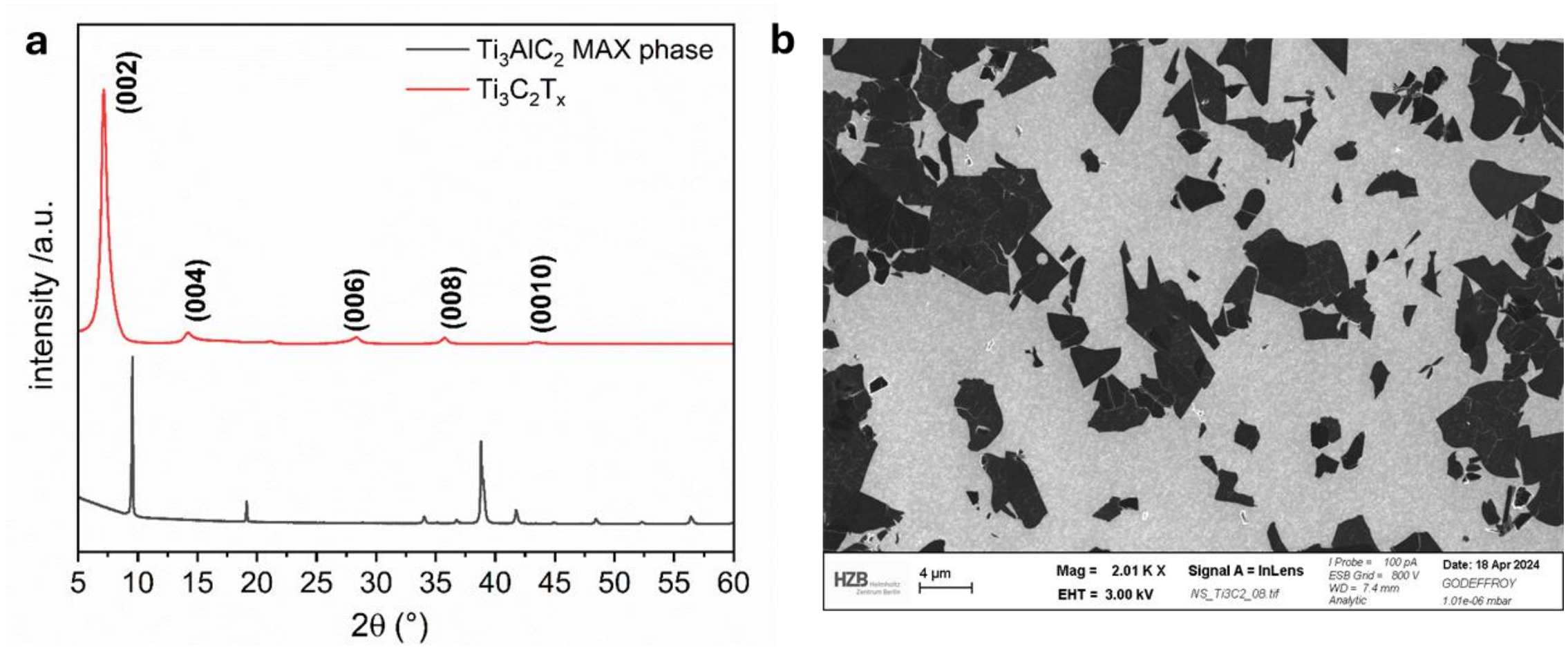

Supplementary Fig. 23. XRD pattern and SEM following synthesis. (a) X-ray diffraction patterns of the $Ti_3C_2T_x$ MXene used in this work (red) and of the Ti_3AlC_2 MAX phase used as precursor for its synthesis (black). (b) Scanning electron micrograph of the $Ti_3C_2T_x$ MXene (drop-cast on an indium tin oxide, ITO, substrate). Source data for the graphs presented in (a) are provided in the Source Data file.

Supplementary References

1. Cockreham, C. B. *et al.* Energetic Stability and Interfacial Complexity of Ti3C2Tx MXenes Synthesized with HF/HCl and CoF2/HCl as Etching Agents. *ACS Appl. Mater. Interfaces* **14**, 41542–41554 (2022).
2. Nayak, P. *et al.* Single-entity Ti3C2Tx MXene electro-oxidation. *Appl. Mater. Today* **26**, 101335 (2022).
3. Lorencova, L. *et al.* Electrochemical performance of Ti3C2Tx MXene in aqueous media: towards ultrasensitive H2O2 sensing. *Electrochim. Acta* **235**, 471–479 (2017).
4. Brunet Cabré, M. *et al.* Isolation of pseudocapacitive surface processes at monolayer MXene flakes reveals delocalized charging mechanism. *Nat. Commun.* **14**, 1–7 (2023).
5. Dessoliers, Z. *et al.* Combining X-Ray Photoelectron and Absorption Spectroscopies for Determining Surface Chemistry and Composition of Ti3C2Tx MXene. *Adv. Mater. Interfaces* **12**, 2500391 (2025).
