# Supplementary material for: Correlative nanoscale chemical imaging reveals delocalized Faradaic processes at isolated MXene flakes
Source: Nat Commun. 2026 Nov 9;17:11330. doi: 10.1038/s41467-026-79237-7 (PMC13748615; doi:10.1038/s41467-026-79237-7)
Supplement: Supplementary file 2 [file 41467_2026_79237_MOESM2_ESM.pdf]

nature portfolio

Peer Review File

Correlative Nanoscale Chemical Imaging Reveals Delocalized Faradaic Processes at Isolated MXene Flakes

Corresponding Author: Dr Tristan Petit

This file contains all reviewer reports in order by version, followed by all author rebuttals in order by version.

Version 0:

Reviewer comments:

Reviewer #1

(Remarks to the Author)
In this manuscript, the authors use scanning X-ray microscopy (SXM) to correlate the electronic structure of $Ti_3C_2T_x$ MXene flakes at the nanoscale with local electrochemical reactions triggered at the sub-flake level by scanning electrochemical cell microscopy (SECCM). The authors demonstrated the delocalization characteristics of the reaction and evidence of hydrogen storage. Overall, this work possesses considerable innovation and research value, but further revisions are still needed. Specific comments are as follows:
1.The authors' description in the introduction is somewhat cumbersome. Although it provides a good overview of the current research status and issues of MXene flakes, it weakens the main research objectives of this study and the logic of the manuscript. This makes it difficult for readers to clearly grasp the research objectives. It is recommended to further refine the introduction section.
2.The author has developed a novel imaging scheme that effectively observes the evolution behavior of single-layer MXene flakes. However, there is a lack of description regarding the specific advantages of this scheme. For instance, comparisons with other current schemes could be included to enhance the innovativeness of the manuscript.
3.Please provide a more detailed explanation of the criteria used to select the three locations shown in Figure 2a.
4.The author has effectively observed that thinner MXene flakes are more prone to spontaneous oxidation than thicker ones. Is it possible to propose a reasonable mechanistic explanation for this phenomenon? Doing so would significantly enhance the scientific value of the manuscript.
5.The author has developed a completely new sample-holding mold. Does this mold have the potential to be used for in-situ electrochemical experiments? Please also identify the unique innovative aspects of this mold.
6.The author should address some issues regarding text formatting; for example, the paragraph “To gain statistical insight into this anodic…on a pixel-by-pixel basis” appears to be noticeably misaligned with the surrounding text. Additionally, the author has arranged the individual subfigures within the figure in a haphazard manner.

Reviewer #2

(Remarks to the Author)
This manuscript combines scanning electrochemical cell microscopy (SECCM) with scanning X-ray microscopy (SXM) to investigate electrochemical activity at individual $Ti_3C_2T_x$ MXene flakes. The authors report that both anodic and cathodic processes extend well beyond the local SECCM contact area and attribute this to delocalized processes mediated by interfacial water. Overall, the manuscript is scientifically significant and well organized with clear and compelling results. However, several aspects require clarification and additional analysis to strengthen the conclusions. The specific comments should be addressed before publication:

1.The main concern is the absence of quantitative analysis of the spatial extent of reaction propagation. While SXM maps show extended oxidation regions, the discussion remains largely qualitative. No propagation length from the SECCM contact point center is provided. The factors governing propagation length are not discussed. Given that the key claim relies on spatial delocalization, such quantification is significant essential. I would suggest the authors to perform a control experiment using a single SECCM contact point at the center of a MXene flake to extract a radial propagation profile. This would also help determine whether the propagation is isotropic or anisotropic within the MXene plane.

2.The SECCM configuration in Figure 1 is very confused. The transparent region in the center of the substrate appears similar a liquid cell. In addition, the “droplet cell residue” is not defined in the caption. I would suggest clarifying the schematic and explicitly define all features in the figure caption. In addition, including the details of the estimated contact area of the droplet cell residue and the area of each grid point would greatly improve readability.

3.The manuscript employs the TEY-SXM to probe the surface chemical signatures of $Ti_3C_2T_x$ MXene flakes. Given that $Ti_3C_2T_x$ contains three Ti layers, it is unclear whether the Ti L-edge signal shown in Figure 2 arises from only the top Ti surface layer or all three Ti layers. Provide a discussion on the probing depth of the technique would be helpful.

4.A key control experiment is missing: TEY-SXM of MXene flakes before the electrochemical reactions for both the anodic and cathodic cycling. Without this baseline, it is difficult to fully attribute the observed oxidation to electrochemical reactions rather than pre-existing or spontaneous oxidation.

5.In Figure 2e, the region above the labeled “Pristine area” are oxidized but is not discussed. Is this region part of the same flake or different flake? Further clarification is needed.

6.Previous work (Fang et al., PNAS, 2024 https://www.pnas.org/doi/10.1073/pnas.2400084121) shows $Ti_3C_2T_x$ MXene edges are more prone to thermal oxidation due to the dangling bonds and exhibit uniform behavior. In contrast, the current manuscript does not show clear edge-dominated oxidation. For example, in the “Oxidized area” of Figure 2e, the top edge is more oxidized than that of the bottom edge, and the right edge fully covered by the SECCM contact points is less oxidized. Provide a more detailed discussion regarding the edge oxidation will improve the quality of the work.

7.The work (Fang et al., PNAS, 2024 https://www.pnas.org/doi/10.1073/pnas.2400084121) suggests that the oxidized products are TiO_2 nanoparticles. However, in the current manuscript the oxidation is interpreted as forming TiO_2 layers. How to confirm that is due to TiO_2 formation not the O-terminations increasing cause the Ti L2 eg/t2g intensity increasing. Is it possibly to quantify the oxidation degree (partially or fully oxidized) based on the intensity ratio change of either Ti L2/L3 or L2 eg/t2g?

8.Previous work (Fang et al., PNAS, 2024 https://www.pnas.org/doi/10.1073/pnas.2400084121) have shown that interlayer confined water drives oxidation in mulflakes MXene, while monoflakes are more thermally stable. In contrast, this manuscript reports that thinner flakes are more susceptible to spontaneous oxidation. Please comments the oxidation mechanism if it is not the water.

9.In the Cathodic Cycling section, multiple processes (HER, proton adsorption and electrical double layer charging) are coupled together. This complicates interpretation of the delocalization phenomenon. To investigate each of process effect on the delocalized process, it is better to investigate them individually. For example, performing the measurements in potential window (-0.3 V to 0.5 V) to isolate protonation and deprotonation process, which could help understand how the surface termination effect on the delocalization. While this work mainly focuses on the delocalized Faradaic processes, including the non-Faradic conditions (e.g., neutral electrolye such as Li_2SO_4, as demonstrated by Zhang et al., Nat Energy 2023 https://www.nature.com/articles/s41560-023-01240-9) would help determine whether delocalization is specific to Faradaic processes or also present in non-Faradaic charging.

10.The scan rate used for cyclic voltammetry should be clearly stated in the figure captions.

Reviewer #3

(Remarks to the Author)
This manuscript reports a correlative SECCM–SXM study of individual $Ti_3C_2T_x$ MXene flakes and addresses an interesting question concerning the intrinsic electrochemical behaviour of MXenes at the single-flake level. The combination of localized electrochemical probing with nanoscale chemical imaging is technically appealing, and the observation that anodic oxidation, capacitive charging and hydrogen-related processes extend beyond the nominal SECCM contact area is interesting. The work is potentially valuable for the broader understanding of electrochemical processes in 2D materials. However, while the experimental observations are compelling, some of the mechanistic conclusions are presently stronger than what is directly supported by the data. In particular, the manuscript would benefit from a clearer distinction between experimentally established observations and mechanistic interpretations. I therefore find the study promising, but minor revision is needed before publication.

1. The manuscript provides convincing evidence that the electrochemical response extends beyond the area directly contacted by the SECCM droplet. However, the interpretation that this behaviour originates from ultrafast lateral proton transport across the MXene surface or through its interlayers is not yet fully demonstrated. At present, the data support the existence of non-local electrochemical activity, but they do not unambiguously distinguish between intrinsic proton transport within the MXene and other possible contributions, such as ionic transport through a very thin electrolyte or water layer extending beyond the visible droplet, or electronic conduction through the flake coupled to reactions occurring away from the contact point. The authors should therefore moderate the mechanistic language or provide additional discussion clarifying why alternative explanations are less likely.

2. The discussion regarding the role of interfacial water is interesting, but the current evidence remains somewhat indirect. The O K-edge measurements and the loss of cathodic response in a subset of flakes are consistent with local water

depletion contributing to the observed behaviour. However, these observations could also reflect irreversible changes in surface chemistry during cycling. Since the manuscript places considerable emphasis on interfacial water as a central factor controlling the delocalized response, this point should be discussed with greater caution. It would be helpful if the authors more clearly separated what is directly observed from what is inferred.

3. The interpretation of the cathodic features in terms of hydrogen adsorption/desorption and hydrogen storage is plausible and supported by comparison with previous literature, but it remains indirect. The high pseudocapacitive values and the observed redox peaks are certainly suggestive, yet they do not uniquely establish hydrogen storage as opposed to other proton-coupled surface processes. I would therefore encourage the authors to soften the wording in this section, unless additional evidence is available to support a more definitive assignment.

Version 1:

Reviewer comments:

Reviewer #1

(Remarks to the Author)
The revised manuscript can be accepted in NC.

Reviewer #2

(Remarks to the Author)
The authors have adequately addressed my concerns, and I have no further comments. The manuscript is suitable for publication in its current form.

Reviewer #3

(Remarks to the Author)
I have reviewed the revised manuscript and the authors' responses. All my previous concerns have been satisfactorily addressed. I recommend acceptance of the manuscript in its present form.

Open Access This Peer Review File is licensed under a Creative Commons Attribution 4.0 International License, which permits use, sharing, adaptation, distribution and reproduction in any medium or format, as long as you give appropriate credit to the original author(s) and the source, provide a link to the Creative Commons license, and indicate if changes were made.
In cases where reviewers are anonymous, credit should be given to 'Anonymous Referee' and the source.
The images or other third party material in this Peer Review File are included in the article's Creative Commons license, unless indicated otherwise in a credit line to the material. If material is not included in the article's Creative Commons license and your intended use is not permitted by statutory regulation or exceeds the permitted use, you will need to obtain permission directly from the copyright holder.
To view a copy of this license, visit https://creativecommons.org/licenses/by/4.0/

Reviewer #1 (Remarks to the Author)

In this manuscript, the authors use scanning X-ray microscopy (SXM) to correlate the electronic structure of Ti3C2Tx MXene flakes at the nanoscale with local electrochemical reactions triggered at the sub-flake level by scanning electrochemical cell microscopy (SECCM). The authors demonstrated the delocalization characteristics of the reaction and evidence of hydrogen storage. Overall, this work possesses considerable innovation and research value, but further revisions are still needed. Specific comments are as follows:

We thank the reviewer for the careful reading of the manuscript and positive comments.

R1.1 The authors' description in the introduction is somewhat cumbersome. Although it provides a good overview of the current research status and issues of MXene flakes, it weakens the main research objectives of this study and the logic of the manuscript. This makes it difficult for readers to clearly grasp the research objectives. It is recommended to further refine the introduction section.

Following the reviewer's suggestion we have revised the introduction to minimise repetition and highlight the main findings.

Changes to manuscript

Changes in the introduction are shown in the highlighted version of the revised manuscript.

R1.2 The author has developed a novel imaging scheme that effectively observes the evolution behavior of single-layer MXene flakes. However, there is a lack of description regarding the specific advantages of this scheme. For instance, comparisons with other current schemes could be included to enhance the innovativeness of the manuscript.

Following the reviewer's suggestion we have explicitly referenced our prior manuscript describing the methodology and that provides a detailed comparison to other correlative imaging approaches in the literature. In a previous study, we had investigated the capacitive response of MXenes (ref. 9) using correlative SECCM and SEM, which could only suggest that delocalization plays a role in the observed response. Combining SECCM with SXM instead, which has recently demonstrated great potential for MXene research (e.g., ref. 26 and ref. 29), we were able to monitor local changes of Ti bonding environment at the sub-flake level and actually provide first evidence for these delocalized phenomena.

Changes to manuscript

In p. 2 we refer the reader to prior work that managed to probe the capacitive response of single MXene flakes and detailing the development of the SECCM-SXM correlative method and in

p.3 we have amended the text to how the reactions have been broadened the reactions to include HER as well as MXene oxidation.

R1.3 Please provide a more detailed explanation of the criteria used to select the three locations shown in Figure 2a.

Locations probed via SECCM were identified via SXM but could not be chosen in advance, as the SECCM and SXM characterizations cannot be carried out simultaneously. SECCM was carried out first on drop cast samples; afterwards, SXM imaging of probed locations was carried out, as originally described in the main text for Figure 1. Therefore, the electrochemical analysis of anodic processes could be focused on MXene flakes that were isolated (not overlapping with other MXenes) and for which the transmission images allowed for thickness estimates (monolayer vs multilayer), which was essential for relating the total anodic charge to the chemical contrast in Figure 2.

Changes to manuscript

We have revised the experimental section (p. 14) to explicitly indicate that grids were positioned at first and only later characterized via SXM, as shown in the highlighted version of the manuscript.

R1.4 The author has effectively observed that thinner MXene flakes are more prone to spontaneous oxidation than thicker ones. Is it possible to propose a reasonable mechanistic explanation for this phenomenon? Doing so would significantly enhance the scientific value of the manuscript.

Oxidation of MXene is mostly induced by water molecules from ambient air, to which the top MXene layer is more exposed to. In the case of multilayers, the top layer is protecting the layers below from moisture and the MXene flake therefore appears less oxidized. This result was discussed in our previous work (ref. 26) and was also confirmed by the lower conductivity of 1–3-layer thin MXene flakes compared to thicker ones (ref. 36). Over extended exposure to humid air or extended storage in aerated dispersions (days), some water molecules will still end up intercalating between the layers of multilayer MXenes, starting from the edges, leading to more oxidized edges as observed in several studies (e.g., 10.1016/j.apsusc.2020.147157). However, this does not significantly affect the average oxidation state of the flakes.

Changes to manuscript:

On p. 7 we have revised the manuscript to indicate that the higher proportion of surface Ti atoms exposed to air in monolayer flakes is what likely leads to more prominent spontaneous oxidation in thin flakes.

R1.5 The author has developed a completely new sample-holding mold. Does this mold have the potential to be used for in-situ electrochemical experiments? Please also identify the unique innovative aspects of this mold.

We thank the reviewer for this suggestion. It would not be possible to carry out in situ SECCM-SXM, as also mentioned in our response to R1.3, due to the probing geometry and hindrances of the SECCM nanopipette. Even if it was possible to place to nanopipette at an angle so that it would not be blocking the X-ray path, SXM, especially in TEY mode, needs to be operated under ultra-high vacuum, which makes "open" electrochemical measurements de facto impossible. However, it is still possible to carry out *in situ* electrochemistry in combination with SXM in transmission, as shown by Sharma, N. *et al.* (ref. 29), using confined cells that isolate the liquid from the vacuum. In this case, a standard three-electrode cell is deposited directly on the SiN_x membrane and allows *in situ* electrochemistry. The current manuscript is complementary to ref. 29 as it is not possible to expose only local areas of the MXene flake with the *in situ* electrochemical cell; the entire flake is polarized and electrolytically bridged, hindering the observation of such delocalized processes.

R1.6 The author should address some issues regarding text formatting; for example, the paragraph "To gain statistical insight into this anodic…on a pixel-by-pixel basis" appears to be noticeably misaligned with the surrounding text. Additionally, the author has arranged the individual subfigures within the figure in a haphazard manner.

We thank the reviewer for their comment.

Changes to manuscript

We have carefully proofread the manuscript and made the formatting of paragraphs and indents uniform throughout.

Reviewer #2 (Remarks to the Author)

This manuscript combines scanning electrochemical cell microscopy (SECCM) with scanning X-ray microscopy (SXM) to investigate electrochemical activity at individual Ti3C2Tx MXene flakes. The authors report that both anodic and cathodic processes extend well beyond the local SECCM contact area and attribute this to delocalized processes mediated by interfacial water. Overall, the manuscript is scientifically significant and well organized with clear and compelling results. However, several aspects require clarification and additional analysis to strengthen the conclusions. The specific comments should be addressed before publication:

We thank the reviewer for their careful reading of the manuscript and positive comments. We appreciate each of their suggested inputs and revisions that are answered below point by point.

R2.1 The main concern is the absence of quantitative analysis of the spatial extent of reaction propagation. While SXM maps show extended oxidation regions, the discussion remains largely qualitative. No propagation length from the SECCM contact point center is provided. The factors governing propagation length are not discussed. Given that the key claim relies on spatial delocalization, such quantification is significant essential. I would suggest the authors to perform a control experiment using a single SECCM contact point at the center of a MXene flake to extract a radial propagation profile. This would also help determine whether the propagation is isotropic or anisotropic within the MXene plane.

We agree with the reviewer that precise control over the nanopipette probe positioning relative to the flake area would be ideal. However, this would require sub-micron resolution mapping of the flake's position over a macroscopic area prior to SECCM measurements, as well as accurate correlation of this position with that of the nanopipette probe, an approach that is currently not feasible in our setup. This could be done via SEM or SXM; however, e-beam/X-ray exposure could also potentially alter the MXenes prior to SECCM probing. By probing first via SECCM, we ensure that the electrochemical response measured is that of a freshly drop-cast pristine nanomaterial. More importantly, to interpret the response to electrochemical stimuli, it is essential to also control thickness/stacking of the flake under study. This requires SXM transmission measurements in advance of SECCM measurements, which makes it logistically unfeasible for typical beamtime allocations. Our analysis is instead done retrospectively, where we can quantitatively measure charge for those flakes probed via SECCM for which both area and thickness can be determined based on SXM maps. Over the course of the two experiments conducted for this manuscript (the anodic and cathodic electrochemical probing), we contacted a total of 19 flakes. Of these 19, 7 were contacted only once. Unfortunately, within this dataset, the single contacted flakes are more than one layer thick or lack sufficient SXM data that would allow measuring a radial propagation profile relating contact position to oxidation of Ti.

Still, the flake in Figure 2 allows to estimate the radial propagation length if we consider the first contact negligible in terms of charge: it is approximately 5 µm. However, given that

complete information on electrochemical response, thickness and surface oxidation state is only available for a small sub-set of flakes, we prefer not to give any numbers in the main as they might be misleading. If we go even further in the interpretation, assuming the reaction occurs over the 1.0-1.2 V window in each scan, then the reaction time is approximately 1.2 s, meaning the diffusion coefficient should be $\approx 2 \times 10^{-11}$ m^2 s^{-1}. This is consistent with the fact that protons are not diffusing in bulk like water ($D_{bulk} = 9 \times 10^{-9}$ m^2 s^{-1}) but probably in an extremely thin layer of water on top of the MXene surface.

Based on this simple model, we hypothesize that scan rate, by modulating the reaction time, and humidity, by modulating the thickness of the surface water layer and hence the diffusion coefficient (going towards bulk values for larger thicknesses), should be the most important parameters in controlling the propagation length. We are currently working on implementing *operando* optical microscopy measurements to track the propagation of the oxidation front in real time, but these measurements are out of the scope of this study where we rather aim at providing precise information about the composition and electronic structure of locally electrochemically probed MXene flakes to evidence these delocalized electrochemical phenomena.

R2.2 The SECCM configuration in Figure 1 is very confused. The transparent region in the center of the substrate appears similar a liquid cell. In addition, the "droplet cell residue" is not defined in the caption. I would suggest clarifying the schematic and explicitly define all features in the figure caption. In addition, including the details of the estimated contact area of the droplet cell residue and the area of each grid point would greatly improve readability.

We thank the reviewer for their comment and suggestions.

Changes to manuscript

We have revised the Figure 1 in the manuscript (p. 4) to include an indication that the aperture is composed of SiN_x so to not confuse it with a liquid cell. We have also revised the caption for Figure 1 in the manuscript (p. 4) and adapted it to define the droplet cell, the droplet cell residue and its size.

R2.3 The manuscript employs the TEY-SXM to probe the surface chemical signatures of Ti3C2Tx MXene flakes. Given that Ti3C2Tx contains three Ti layers, it is unclear whether the Ti L-edge signal shown in Figure 2 arises from only the top Ti surface layer or all three Ti layers. Provide a discussion on the probing depth of the technique would be helpful.

The probing depth at the Ti L-edge is in the order of 2-4 nm (see ref. 26), therefore all the three Ti layers are probed by SXM. The probing depth has been clarified in the revised manuscript.

Changes to manuscript

Page 3: The TEY-SXM mode is particularly advantageous for characterizing surface reactions due to its shallow probing depth of 2-4 nm at the Ti L-edge, mostly probing the first MXene layer (3 Ti layers), compared to the bulk-sensitive transmission mode (STXM).[26]

R2.4 A key control experiment is missing: TEY-SXM of MXene flakes before the electrochemical reactions for both the anodic and cathodic cycling. Without this baseline, it is difficult to fully attribute the observed oxidation to electrochemical reactions rather than pre-existing or spontaneous oxidation.

We agree with the reviewer that this would be ideal, however carrying out SXM transmission measurements in advance of SECCM measurements is not feasible for typical beamtime allocations considering that SECCM and SXM are not carried out simultaneously but in two geographically distinct locations. Further, as explained in the answer to comment R2.1, X-ray beam exposure can also potentially alter or contaminate the MXenes prior to SECCM probing. By probing first via SECCM, we ensure that the electrochemical response measured is that of a freshly drop-cast nanomaterial.

The workflow as detailed in the manuscript (p. 3-4) and in R1.2 describes the drop cast of the samples, followed by their immediate SECCM measurements and then their subsequent SXM measurements. It is therefore not possible to have SXM measurements of the samples prior to the SECCM measurements. However, this work does showcase data from an unprobed flake. Figure 3 includes data from this unprobed flake, and it is depicted in Figure S11.

Our prior work by Amargianou, F. *et al.* (ref. 26) focused on a detailed characterization of freshly synthesized MXenes, which addresses the reviewer's comment. In this revised version (p. 7), we therefore include a comparison of oxidation state of electrochemically probed MXenes vs that of unprobed MXenes, in addition to showcasing data for an unprobed flake.

R2.5 In Figure 2e, the region above the labeled "Pristine area" are oxidized but is not discussed. Is this region part of the same flake or different flake? Further clarification is needed.

This region is most likely coming from a different flake partially below the main flake, as Figure 2a and e suggest. In the OD image, there is a larger contrast so a thicker area where both flakes overlap, clearly showing these are two distinct flakes. It is however unclear why this flake is more oxidized, but we do not think that it is relevant to discuss it in the main manuscript as it is not related to the flake in contact with the electrolyte. We often see smaller fragments being more oxidized than larger flakes like our flake of interest.

In addition, we see in Figure 2e that the region where the two flakes overlap shows a low oxidation state so it is clear that the large flake's response is not affected by that of the small fragment.

R2.6 Previous work (Fang et al., PNAS, 2024 https://www.pnas.org/doi/10.1073/pnas.2400084121) shows Ti3C2Tx MXene edges are more prone to thermal oxidation due to the dangling bonds and exhibit uniform behavior. In contrast, the current manuscript does not show clear edge-dominated oxidation. For example, in the "Oxidized area" of Figure 2e, the top edge is more oxidized than that of the bottom edge, and the right edge fully covered by the SECCM contact points is less oxidized. Provide a more detailed discussion regarding the edge oxidation will improve the quality of the work.

We agree that edges are more prone to spontaneous oxidation due to defects and we have also imaged this using SXM in our previous work (ref. 26). We believe the fact that the bottom and right edges of the flake in Figure 2e appear less oxidized is rather linked to the radial propagation of the oxidation front from the second contact point as discussed in comment R2.1 and represented in the scheme below. As explained in the article (p. 6), the first contact point, even if it is very close to these edges, yielded very little charge and therefore probably did not lead to a significant oxidation front. In general, the oxidation is here driven electrochemically from the basal plane, so the edge defects do not, in our mind, play a significant role. All the more so since the electrochemical oxidation is anyways leading to the highest possible oxidation state of Ti i.e., +4.

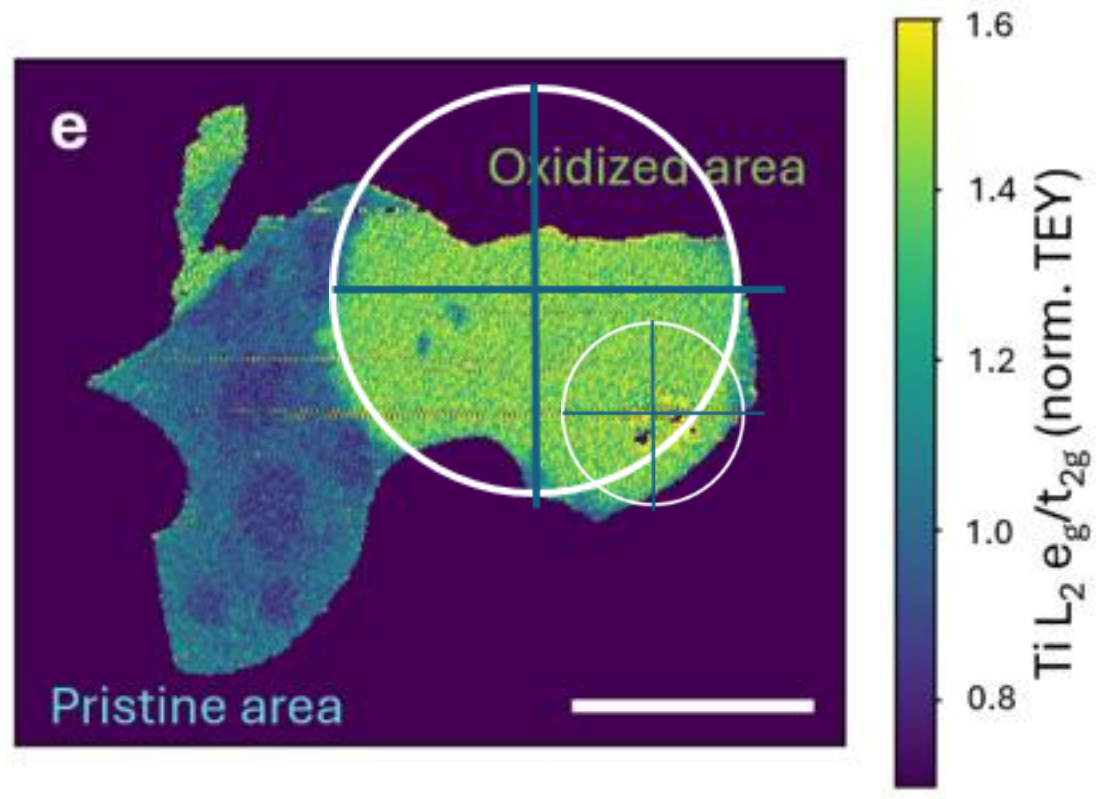

Figure R1: Figure 2e with overlaid circles centred at the SECCM contact points. The circles illustrate the radial propagation of the oxidation front and clarify the lower oxidation state observed in some edge portions of the southern and eastern regions of the oxidized area.

R2.7 The work (Fang et al., PNAS, 2024 https://www.pnas.org/doi/10.1073/pnas.2400084121) suggests that the oxidized products are TiO2 nanoparticles. However, in the current manuscript the oxidation is interpreted as forming TiO2 layers. How to confirm that is due to TiO2 formation not the O-terminations increasing cause the Ti L2 eg/t2g intensity increasing. Is it possibly to quantify the oxidation degree (partially or fully oxidized) based on the intensity ratio change of either Ti L2/L3 or L2 eg/t2g?

We thank the reviewer for suggesting the cited manuscript. The cited literature indicates that TiO_2 nanoparticles are formed via oxidation of multilayers in air at high temperature (300-400°C). These conditions are significantly different from electrochemical oxidation carried out at room temperature, for which the resulting products are not expected to be affected by sintering and crystallization processes. The intensity ratio of the L2 eg/t2g can indeed be used to estimate the Ti oxidation state, as detailed in ref. 29. The average oxidation state is therefore 4.4 vs 3.1 in the pristine area, which is in good agreement with TiO_2. Note that we have also recorded XAS at the carbon K-edge (Figure S10), showing that the carbide structure is lost after electrochemical oxidation.

Changes to manuscript

P. 4 of the revised manuscript refers the reader to estimates of oxidation state based on calibration of e_g/t_{2g} ratios, discussed in ref. 29 in greater detail.

P. 6 now includes specific mention to the similarities between anodic oxidation and air oxidation conditions in the cited literature and the work by Fang et al. has been now added to the references.

R2.8 Previous work (Fang et al., PNAS, 2024 https://www.pnas.org/doi/10.1073/pnas.2400084121) have shown that interlayer confined water drives oxidation in mulflakes MXene, while monoflakes are more thermally stable. In contrast, this manuscript reports that thinner flakes are more susceptible to spontaneous oxidation. Please comments the oxidation mechanism if it is not the water.

We agree that water is the main source of oxidation. In our experiment, carried out on freshly drop cast MXene flakes and under ambient conditions, the top layer is the most exposed to moisture. In the work of Fang *et al.*, adsorbed water on the top layers is removed at low temperature during annealing, therefore the effect of confined water is enhanced at high temperature. In the case where the sample is fully immersed in water, thin flakes where also found to be more oxidized (ref. 29). Therefore, our observations are consistent with the argument that water facilitates oxidation; however, it is the difference in experimental configuration that results in the greater propensity of monolayers towards oxidation. Comparing the two approaches is very insightful, as it shows that adsorbed water on the top layers is really the key player in driving oxidation in aqueous solutions.

Changes to manuscript

P. 6 of the manuscript now refers the reader to the similarities between thermal and electrochemical oxidation of MXenes.

R2.9 In the Cathodic Cycling section, multiple processes (HER, proton adsorption and electrical double layer charging) are coupled together. This complicates interpretation of the delocalization phenomenon. To investigate each of process effect on the delocalized process, it is better to investigate them individually. For example, performing the measurements in potential window (-0.3 V to 0.5 V) to isolate protonation and deprotonation process, which could help understand how the surface termination effect on the delocalization. While this work mainly focuses on the delocalized Faradaic processes, including the non-Faradic conditions (e.g., neutral electrolye such as Li2SO4, as demonstrated by Zhang et al., Nat Energy 2023 https://www.nature.com/articles/s41560-023-01240-9) would help determine whether delocalization is specific to Faradaic processes or also present in non-Faradaic charging.

We thank the reviewer for suggesting the manuscript by Zhang *et al.* which studies MXene films of thickness ~50 nm to ~150 nm. One of the main aims of our work is to disentangle the extrinsic response of MXenes (arising from a combination of intrinsic properties and the overall morphology of film/electrode, e.g. pore volume and percolation networks) from the intrinsic one arising from a single flake (of well-defined thickness). Indeed, the use of porous 3D electrodes can often confound the interpretation of the electrochemical response, whereby effects arising from electrode morphology are attributed to changes in the intrinsic surface response. This is a well-known problem in the literature of 2D materials such as graphene oxide (demonstrated by Kaliyaraj Selva Kumar *et al.*, *ACS Catal.* 2022, 12, 4754). Our manuscript reports for the first time a characterization of the faradaic response of isolated 2D MXenes without confounding effect arising from intra film mass transport limitations.

While performing similar measurement in Li_2SO_4 might indeed be insightful, the non-faradaic response will likely be very different from the one measured in H_2SO_4 and therefore not really allow to disentangle it from the faradaic response as suggested by the reviewer. While $Ti_3C_2T_x$ usually behaves like a pure capacitor in Li_2SO_4, pseudocapacitive processes also take place in H_2SO_4 as discussed in the paper (which are still isolated from the HER/hydrogen storage which is a purely faradaic process). Hence, we believe that while these experiments could indeed be very insightful, they are out of the scope of this study.

R2.10 The scan rate used for cyclic voltammetry should be clearly stated in the figure captions.

We thank the reviewer for this suggestion and have implemented this change into the manuscript.

Changes to manuscript

All measurements (both anodic and cathodic experiments) were carried out at 500 mV/s. Each of the captions in the main manuscript and the supporting information now includes this information.

Reviewer #3 (Remarks to the Author):

This manuscript reports a correlative SECCM–SXM study of individual Ti3C2Tx MXene flakes and addresses an interesting question concerning the intrinsic electrochemical behaviour of MXenes at the single-flake level. The combination of localized electrochemical probing with nanoscale chemical imaging is technically appealing, and the observation that anodic oxidation, capacitive charging and hydrogen-related processes extend beyond the nominal SECCM contact area is interesting. The work is potentially valuable for the broader understanding of electrochemical processes in 2D materials. However, while the experimental observations are compelling, some of the mechanistic conclusions are presently stronger than what is directly supported by the data. In particular, the manuscript would benefit from a clearer distinction between experimentally established observations and mechanistic interpretations. I therefore find the study promising, but minor revision is needed before publication.

We thank the reviewer for their careful reading of the manuscript and positive comments. We appreciate each of their suggested inputs and revisions that are answered below point by point.

R3.1. The manuscript provides convincing evidence that the electrochemical response extends beyond the area directly contacted by the SECCM droplet. However, the interpretation that this behaviour originates from ultrafast lateral proton transport across the MXene surface or through its interlayers is not yet fully demonstrated. At present, the data support the existence of non-local electrochemical activity, but they do not unambiguously distinguish between intrinsic proton transport within the MXene and other possible contributions, such as ionic transport through a very thin electrolyte or water layer extending beyond the visible droplet, or electronic conduction through the flake coupled to reactions occurring away from the contact point. The authors should therefore moderate the mechanistic language or provide additional discussion clarifying why alternative explanations are less likely.

We understand the point of the reviewer and agree to moderate the language about the mechanism.

Changes to manuscript

Changes in the main text, mostly in the discussion and conclusion parts on p. 12, are shown in the highlighted version of the revised manuscript.

R3.2. The discussion regarding the role of interfacial water is interesting, but the current evidence remains somewhat indirect. The O K-edge measurements and the loss of cathodic response in a subset of flakes are consistent with local water depletion contributing to the observed behaviour. However, these observations could also reflect irreversible changes in surface chemistry during cycling. Since the manuscript places considerable emphasis on interfacial water as a central factor controlling the delocalized response, this point should be

discussed with greater caution. It would be helpful if the authors more clearly separated what is directly observed from what is inferred.

We thank the reviewer for highlighting this point. The excitation energy at the O K-edge of 535.5 eV was selected to precisely excite the water contribution and not the MXene surface groups, which occurs between 530-534 eV (see spectra in Figure R2 below and spectra of pristine MXene films in Figure S10). We are therefore confident that the contrast change at this energy is related to water depletion rather than surface chemistry.

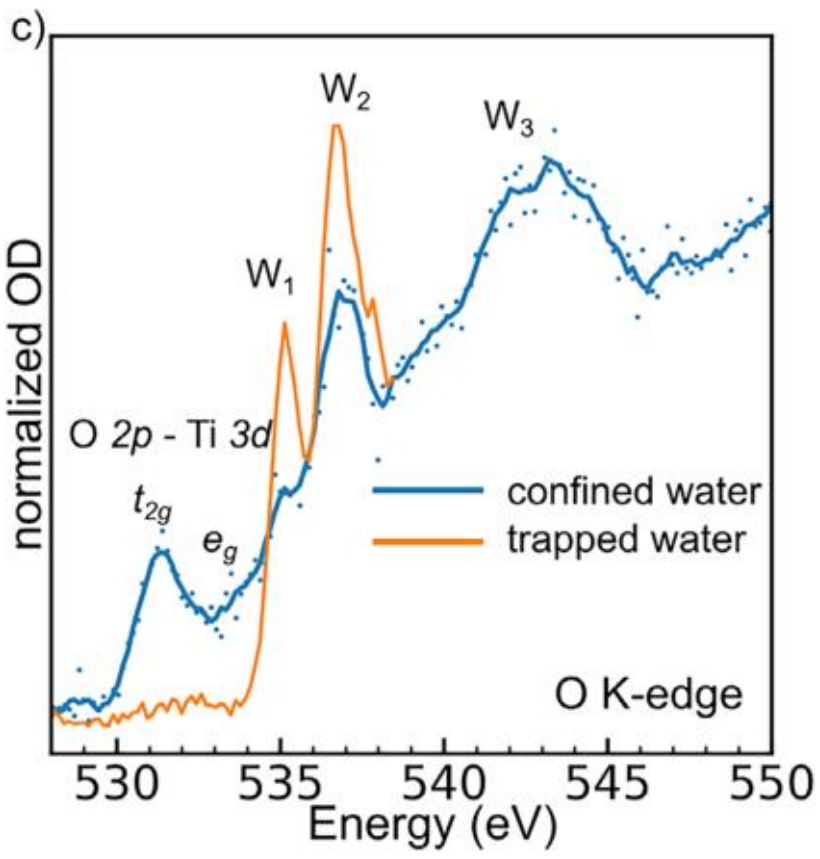

Figure R2: XAS spectra at the O K-edge, acquired for water trapped between overlapping MXene flakes (orange) and confined within the basal plane of a few-layered $Ti_3C_2T_x$ MXene flake (blue). Adapted from ref. 30.

Changes to manuscript

We have clarified this point in the revised manuscript, p. 10: "According to reference spectra of MXene electrodes in **Figure S10**, this feature does not correspond to changes in the MXene's surface chemistry. Instead, it has previously been attributed to the pre-edge of the electronic signature of water molecules.[30,48]"

R3.3. The interpretation of the cathodic features in terms of hydrogen adsorption/desorption and hydrogen storage is plausible and supported by comparison with previous literature, but it remains indirect. The high pseudocapacitive values and the observed redox peaks are certainly suggestive, yet they do not uniquely establish hydrogen storage as opposed to other proton-coupled surface processes. I would therefore encourage the authors to soften the wording in this section, unless additional evidence is available to support a more definitive assignment.

We thank the reviewer for their suggestion. We agree that the associated peaks along with high capacitance values are indicative of hydrogen sorption being observed, while alternative proton-coupled surface processes cannot be ruled out.

Changes to manuscript

In response to the reviewer's comment, we have revised the wording in this section and the conclusion (p. 12) to adopt a more cautious interpretation. The discussion now emphasizes that the electrochemical behaviour is highly consistent with, and strongly suggests, hydrogen sorption, while avoiding definitive assignment in the absence of direct supporting evidence.
